# Nsite, NsiteH and NsiteM computer tools for studying transcription regulatory elements

**DOI:** 10.1093/bioinformatics/btv404

**Published:** 2015-07-02

**Authors:** Ilham A. Shahmuradov, Victor V. Solovyev

**Affiliations:** ^1^Computer, Electrical and Mathematical Sciences and Engineering Division, KAUST, Thuwal 23955-6900, KSA,; ^2^Bioinformatics laboratory, Institute of Botany, ANAS, Baku AZ1073, Azerbaijan and; ^3^Bioinformatics Division, Softberry Inc., Mount Kisco, NY 10549, USA

## Abstract

**Summary:** Gene transcription is mostly conducted through interactions of various transcription factors and their binding sites on DNA (regulatory elements, REs). Today, we are still far from understanding the real regulatory content of promoter regions. Computer methods for identification of REs remain a widely used tool for studying and understanding transcriptional regulation mechanisms. The *Nsite*, *NsiteH* and *NsiteM* programs perform searches for statistically significant (non-random) motifs of known human, animal and plant one-box and composite REs in a single genomic sequence, in a pair of aligned homologous sequences and in a set of functionally related sequences, respectively.

**Availability and implementation:** Pre-compiled executables built under commonly used operating systems are available for download by visiting http://www.molquest.kaust.edu.sa and http://www.softberry.com.

**Contact:** solovictor@gmail.com

**Supplementary information:** Supplementary data are available at *Bioinformatics* online.

## 1 Introduction

Transcription regulatory elements (REs) bound by transcription factors (TFs) are main players in gene expression (Grünberg and Hahn, 2013). Although a large set of experimentally identified REs/TFs has been collected in several databases (Ramirez and Basu, 2009; Solovyev *et al.*, 2010), the real RE content of promoters of most genes remains unidentified.

Established computational RE identification algorithms are predominantly based on one of two approaches: (i) the search for motifs of known REs or (ii) the comparative analysis of homologous sequences aimed to discover new REs (Ladunga, 2010; Solovyev *et al.*, 2010). The first type of methods uses regulatory site and/or IUPAC consensus sequences or position-weight matrices. One of challenges in RE detection is to estimate the statistical significance of located motifs to distinguish them from random matches. In addition, in some cases, TFs bind a composite RE (a pair of DNA motifs with a spacer sequence of variable length between them) rather than a single short DNA region.

Here, we present *Nsite, NsiteH* and *NsiteM*, a set of programs to predict both single and composite REs in query sequences and estimate their statistical significance.

## 2 Results

Previously, we proposed a probabilistic model that computes the probability of observing given sequence motifs or consensuses in random nucleotide sequences of the same length and nucleotide frequencies as a query sequence. The model also estimates the expected number of such motifs in random sequences. In particular, the model assumes that because REs are small that numbers rather than frequencies of nucleotides should be used to describe RE consensus sequences (Shakhmuradov *et al.*, 1986; Solovyev *et al.*, 2010; see also Supplementary Material S4). These statistical estimations provide the opportunity to find non-random similarities (unlikely to have occurred by chance) between a set of functional motifs and regions of an analyzed sequence. By applying this approach, we developed the *Nsite, NsiteH* and *NsiteM* computer programs that use various functional motif datasets. Using data from the largest three transcription RE databases: *TRANSFAC* (Wingender *et al.*, 2001), *oTFD* (Ghosh, 2000) and *RegSite DB* (http://linux1.softberry.com/berry. phtml?topic=regsite), we composed two animal (ooTFD and TRANSFAC) RE datasets and one plant (RegSite) RE dataset containing 8030, 3486 and 2871 single or composite REs, respectively. Other frequently cited and available sources of plant transcription REs, *PLACE* (http://www.dna.affrc.go.jp/PLACE/info.html) and *PlantCARE* (http://bioinformatics.psb.ugent.be/webtools/plantcare/html/) include much less known REs or their consensuses (469 and 435 records, respectively). A user has a choice of selecting one of three datasets or providing own RE set as well as adjusting the search parameters. The format of RE datasets is presented in Supplementary Figure S1 (Supplementary Material S1).

*Nsite* performs searches for statistically non-random motifs of known REs in a single DNA sequence. A predicted motif is considered as statistically significant whether (i) the expected (by chance) number of such motifs is less than a given threshold and (ii) the total number of identified motifs is ≥95% confidence interval upper limit. The search and statistical estimations are performed separately on both strands of a query sequence.

*NsiteH* discovers RE motifs with a given conservation level in a pair of aligned orthologous (homologous) sequences. Sequences should be aligned beforehand, e.g. using the program SCAN2 (http://softberry.com/scan.html). To run NsiteH, three input files are required (two query sequences and their alignment). In comparison to Nsite, this program identifies functional motifs that demonstrate a certain level of similarity between RE motifs in two query sequences.

*NsiteM* searches for statistically significant RE motifs observed in many homologous sequences. This condition serves as an additional criterion for selecting putative REs. By comparison with Nsite, this program applies one additional search parameter—a minimal portion of query sequences containing the same RE motif. As input data, it requires two or more sequences in FASTA format.

Descriptions of output results of these programs are presented in Supplementary Figures S2–S4 (Supplementary Material S1) and their algorithms are outlined in Supplementary Material S4.

**Fig. 1.**
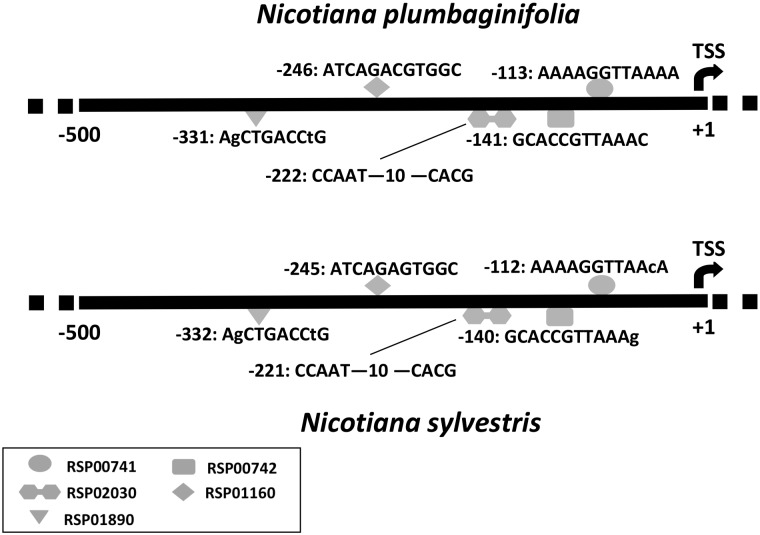
A set of relevant REs predicted by the NsiteH program in proximal promoter regions of orthologous genes Cab-E and Lhcb1*5 encoding chlorophyll a/b-binding protein in *N.plumbaginifolia* and *N.sylvestris.* RSP02030: ERSE-I, RSP01890: W-box. For the full list of predicted REs see the Supplementary Material S2

Testing Nsite, NsiteH and NsiteM on plant and animal sequences indicates that these programs can reliably identify known REs of promoters. For example, applying NsiteH for analysis of promoter regions of the orthologous Cab-E and Lhcb1*5 genes encoding the chlorophyll a/b-binding protein in *Nicotiana plumbaginifolia* and *Nicotiana sylvestris*, we identified a set of evolutionarily conservative REs (Fig. 1). The predicted GT-1 binding sites (RSP00741 and RSP00742) and G-box (CG-1 binding site; RSP01160) are involved in the photoregulation of plant genes and are known to be functional in the Lhcb1*5 gene of *N.plumbaginifolia* (Schindler and Cashmore, 1990).

## 3 Conclusion

The Nsite, NsiteH and NsiteM computer tool for identification of REs in promoter sequences is widely used by researchers, accessible through the Softberry and KAUST Bioinformatics WEB servers (www.softberry.com and www.molquest.kaust.edu.sa), and is cited in ∼200 research articles (according to Google Scholar). Nsite is applied for identification of RE patterns in a single query sequence. Nevertheless, reliable detection of short functional motifs increases when we account for sequence conservation in homologs promoters from different organisms. NsiteH is designed for analysis of orthologous genes’ promoters. NsiteM detects REs involved in the coordinated expression regulation of a group of genes. Our programs provide possibility to search for statistically significant sequence motifs and composite elements. The other analogous consensus-based search tools such as *SIGNAL* SCAN: http://www.dna.affrc.go.jp/sigscan/signal.html*; PlantCARE Search Tool*: http://bioinformatics.psb.ugent.be/webtools/ plantcare/html/; *PatSearch*: http://www.bio.net/bionet/mm/bionews/ 1996-October/ 003416.html search for a single motifs only and do not provide any statistical estimations. There are several studies that experimentally confirmed functionality of RE motifs that were predicted by Nsite program (Delatorre *et al.*, 2012; Linher-Melville and Singh, 2014; Wu *et al.*, 2013; Zheng *et al.*, 2010; Zografidis *et al.*, 2014).

*Conflict of Interest*: none declared.

## Supplementary Material

Supplementary Data
