# Supplementary material for: Nsite, NsiteH and NsiteM computer tools for studying transcription regulatory elements
Source: Bioinformatics. 2015 Jul 2;31(21):3544–5. doi: 10.1093/bioinformatics/btv404 (PMC4612222; doi:10.1093/bioinformatics/btv404)
Supplement: Supplementary Data [file supp_31_21_3544__index.html]

Nsite, NsiteH and NsiteM computer tools for studying transcription regulatory elements — Nsite, NsiteH and NsiteM computer tools for studying transcription regulatory elements — Supplementary Data 

# Nsite, NsiteH and NsiteM computer tools for studying transcription regulatory elements

## Supplementary Data

files

- Supplementary Data - doc file
- Supplementary Data - doc file
- Supplementary Data - docx file
- Supplementary Data - docx file
