# Supplementary material for: Nsite, NsiteH and NsiteM computer tools for studying transcription regulatory elements
Source: Bioinformatics. 2015 Jul 2;31(21):3544–5. doi: 10.1093/bioinformatics/btv404 (PMC4612222; doi:10.1093/bioinformatics/btv404)
Supplement: Supplementary Data [file supp_btv404_BNotes_NSITE_Sovovyev_Supplementary_1.doc]

**Table S1.** Motifs of known REs predicted by the **Nsite** program in the [-500:-1] region upstream of the translation start of the nuclear Lhcb1*5 gene (AC: AB012638.1) encoding chloroplast light-harvesting chlorophyll a/b-binding protein**^1^** in *Nicotiana sylvestris.*

| **Name, Accession Number^2^ and Binding Factor of known RE** | **Organism^3^ and Gene** | **Positions of identified RE motifs ^4^** | **Sequence of identified RE motifs^5^** |
| --- | --- | --- | --- |
| CCA1 BS2 (RSP00629);  CCA1 | At: Lhcb1*3 | -165:-158  -186:-193 | AAAAATCT  AAAAATCT |
| box 1 (RSP00741), GT-1 | Np: Cab-E | -112:-101 | AAAAGGTTAcAA |
| box 2r (RSP00742), GT-1 | Np: Cab-E | -140:-151 | GCACCGTTAAAg |
| G-box (RSP01160), CG-1 | Np: Cab | -245:-234 | ATCAGACGTGGC |
| GBF1 BS (RSP01342), GBF1 | At: Cab-E | -246:-231 | AATCAGACGTGGCAAA |
| half G-box [core]  (RSP00683), GBF3 | At: Adh | -131:-122 | GCCAAaTGGA |
| Gap box 1 (RSP01013), GAPF | At: GapA | -129:-120 | CAAATGGAaA |
| Gap box 2 (RSP01014), GAPF | At: GapA | -197:-206 | CcAATGAAGA |
| Gap box 1 (RSP01019), GAPF | At: GapB | -200:-206 | ATGAAGA |
| ACGT-motif (RSP01831), AREB2/ABF4; GBF3; bHLH080 | At: Cytc-2 | -184:-177 | AAACGTAT |
| EE [TRX m1] (RSP02288), CCA1 | Ps: TRX m1 | -71:-64 | TAGATATT |
| G/A-box (1)  (RSP01718), STF1/HY5 | At: IAA3/SHY2 | -186 :-175 | TTAaACGTATAA |

**^1^**In total, 37 statistically, non-random (a level of homology between known RE and motif of ≥90%; the statistical significance of 95%) motifs of 37 known REs were predicted (see **Supplementary File 2**). Some motifs seem to be functional: **(1)** CCA1 binding site (CCA1 BS2 (RSP00629) as well as GT-1 binding sites box1 (RSP00741) and box 2r (RSP00742) are functional motifs found in promoter regions of the orthologous genes Lhcb1*3 and Cab-E from *A.thaliana* and *N.plumbaginifolia*, respectively. All three of these genes belong to the same light-induced gene family. Moreover, G-box elements are responsive to a variety of environmental stimuli for various genes, including those in the chloroplast. (2) Plant bZIP G-box binding factors are involved in the transcription regulation of photosynthetic genes (Siberil et al., 2001). Motifs of binding sites for these factors (RSP01342: GBF1 BS, RSP00683: half G-box, RSP01831: ACGT-motif) are also present in the promoter region of the Lhcb1*5 gene. **(2)** Chloroplast-related GapA and GapB are induced via Gap boxes and their binding factors GAPF (Conley et al., 1994). The promoter region of the Lhcb1*5 gene contains motifs of GAPF binding sites, Gap box 1 (RSP01013) and Gap box 2 (RSP01014) of the *A. thaliana* GapA gene and Gap box 1 (RSP01019) of the *Arabidopsis* GapB gene. **(3)** CCA1binding Evening Element (EE; RSP02288) is found in the promoter region of the light-regulated chloroplast thioredoxins m1 gene (TRX m1), which mediates the light regulation of carbon metabolism (Barajas-Lopez Jde et al., 2011). Our analysis revealed the motif of this RE upstream of the Lhcb1*5 gene. **(4)** Finally, we demonstrate crosstalk between light and auxin signaling pathways. HY5 and its homolog STF1 play a role in light and hormone signaling pathways. We show that the promoter region of the IAA3/SHY2 gene encoding a repressor of auxin signaling contains HY5/STF1 binding G/A box (RSP01718; Song et al., 2008), evidencing the existence of the G/A box motif in the promoter of the Lhcb1*5 gene.

**^2^** In Regsite (Plant) DB. **^3^** At: *Arabidopsis thaliana*, Np: *Nicotiana plumbaginifolia* and Ps: *Pisum sativum*. **^4^** Positions are given relative to the ATG start codon. **^5^** Low-case letters indicate mismatches.

# Table S2. Putative regulatory elements found upstream of human fibroblast alpha-skeletal actin gene

# (in -707:-1 region located before the annotated pre-mRNA start; gb M20543) using the Nsite program.

| Known RE/Consensus**^a^** | Statistical parameters | | | | Positions of identified motifs | RE motif**^c^** nucleotide sequences,  5’ → 3’ |
| --- | --- | --- | --- | --- | --- | --- |
|  | Maximal allowed and identifiedmismatch  bases | Expected number of motifs**^b^** | 0.95 con-fidence interval | Number of motifs detected |  |  |
| **1** | **2** | **3** | **4** | **5** | **6** | **7** |
| R00034, mouse alpha- actin gene (Binding Factor: hBMAPF2, delta, MAPF2, F-ACT1) | 5 / 0 | 0.000 | 0 | 1 | -102 : -83 | ACACCCAAATATGGCTCGAG |
| R00042, chicken beta-actin gene (ETF) | 5 / 4 | 0.004 | 0 | 1 | --299 : -279 | CcCtgCCCaCCCCAtCCCC |
| R00462, human c-fos gene (SRF) | 2 / 1 | 0.008 | 1 | 1 | -89 : -98 | CCATATTtGG^*^ |
| R00942, human interleukin-2 receptor-alpha gene (NF-kappaB) | 3 / 1 | 0.001 | 1 | 1 | -576 : -588 | aCTCCCTCTCCTT^*^ |
| R01754, human alpha-actin gene, (Sp1) | 1 / 1 | 0.009 | 1 | 2 | -671 : -662  -125 : -116 | GGGGGAaGGG  GaGGGAGGGG |
| R01733, mouse GM-CSF gene (GM-PBP-1) | 3 / 2 | 0.003 | 0 | 1 | -582 : -571 | AGGgAGTTCgCC |
| R02029, human NPY gene (Sp1) | 0 / 0 | 0.000 | 0 | 3 | -282 : -276  -247 : -253  -116 : --122 | CCCCTCC  CCCCTCC^*^  CCCCTCC^*^ |
| R02127, chicken consensus of BGP1 binding element | 2 / 2 | 0.033 | 1 | 1 | -129 : -114 | GGcCGAGGGAGGGGGc |
| R02133, mouse consensus of myc-CF1 binding element  (m-CF) | 0 / 0 | 0.000 | 0 | 4 | -609 : --604  -379 : -374  -94 : --89  -220 : -225 | AAATGG  ACATGG  ATATGG  ATATGG^*^ |
| R02445, human GPC gene (MSN) | 2 / 2 | 0.009 | 0 | 3 | -625 : -615  -597 : --587  -444 : -434 | gGGGGTGGgAA  AGGGGcGtAAA  AGGaGTtGAAA |
| R02657, human apo-CIII gene (COUP,  (HNF-4alpha1,  HNF-4alpha2) | 4 / 4 | 0.003 | 0 | 1 | -306 : --291 | gcGGTGACCcTcGCCC |
| R03000, mouse alpha-actin gene, (myc-CF1) | 5 /2 | 0.000 | 0 | 1 | -101 : -82 | CACCCAAATATGGCTcgAGA |
| R03055, rat *neu* gene (RVF) | 3 / 3 | 0.001 | 0 | 1 | -33 : -20 | CtAtATAAAACCtG |
| R02864, mouse GFAP gene (NF-1) | 5 / 5 | 0.015 | 1 | 1 | -159 : -140 | ggAcCcGGGCgGGGGCCCAG |

# Table S2 (continued)

| **1** | **2** | **3** | **4** | **5** | **6** | **7** |
| --- | --- | --- | --- | --- | --- | --- |
| R03273, rabbit progesterone receptor gene (ERF) | 3 / 2 | 0.007 | 0 | 1 | -662 : -650 | GGTCGACgTGgCT |
| R03356, mouse PGK-1 gene (TIN-1) | 2 / 2 | 0.016 | 0 | 2 | -607 : -598  -582 : -573 | AtGgAGTTCC  AGGgAGTTCg |
| R03496, chicken alpha-actin gene (SRF) | 5 / 5 | 0.001 | 0 | 1 | -184 : -165 | ccGCtCCTTCTTTGGtCAaC |
| R03696, mouse IL-2 gene (AP-3(2), TCF-1,  TCF-2) | 1 / 1 | 0.043 | 0 | 2 | -249 : -240  -192 : -183 | GGGCaAACCC  GGGCcAACCC |
| R04343, human p34 cdc2 gene (c-Ets-2) | 0 / 0 | 0.000 | 0 | 5 | -669 : -664  -619 : -614  -562 : -567 | GGGAAG  GGGAAG  GGGAAG^*^ |

**^a^** ID of known RE or consensus in the TRANSFAC database is indicated.

**^b^** RE motifs with expected mean number of 0.05 or less were searched.

**^c^** RE motifs located on the opposite DNA strand are denoted by an asterisk; mismatches are indicated by lower case letters.

**Table S3.** Putative REs predicted by the **NsiteM** program upstream to the coordinately expressed ribosomal genes of *Saccharomyces cerevisiae* (-900 : -1, region upstream of the AUG initiator codon).

| RE **→** | Binding sites for RAP1**^a^**/RAP1**^b^**/RAP1**^c^**/  RAP1**^d^**/RAP1**^e^** | Binding site for Sp1**^f^** | Binding site for Unknown  Factor**^g^** | Binding site for TIN-1**^h^** | Binding site for Unknown  Factor**^i^** |
| --- | --- | --- | --- | --- | --- |
| Gene (Accession Number in GenBank)  **↓** |
| *BAR1* (YHL015W) | + | + | +++ | ++ | + |
| *rp23* (YNL069C) | ++++ | + | + | + | + |
| *rpl10E* (YLR340W) | +++ | + | + | + | + |
| *NAB1A* (YGR214W) | ++++ | + | + | ++ | + |
| *rpl34B* (YIL052C) | +++++ | + |  | ++ | ++++ |
| *rpL6B* (YLR448W) | +++ | + | +++ | + |  |
| *NAB1B* (YLR048W) | ++++ |  | + | ++++ | + |
| *rpl32* (YGL030W) | +++++++ | + | + | + |  |
| *rpl33B* (YGR034W) | +++++++ | + | + | ++ |  |
| *rps7B* (YJR145C) | +++++++ | + | + |  |  |
| *rpl33A* (YPL143W) | ++++++ | + | + |  | + |
| *rp28B* (YNL301C) | ++ | + | ++ |  |  |
| *TCM1* (YOR063W) | ++ | + | + |  |  |
| *rpl17B* (YER117W) | ++++ |  |  | + | + |
| *rps21* (YOL040C) | ++++ |  | + | +++ | + |
| *rpl5A* (YIL018W) | + | + |  | +++ | + |
| *rps24EA* (YER074W) | ++++ |  |  | + | + |
| *rpl6A* (YGL076C) | ++++ | + |  |  |  |
| *rpl13* (YIL133C) | ++++ | + |  |  |  |
| *SSM1* (YPL220W) | +++ | + |  |  |  |
| *rp55B* (YNL302C) | ++++++ |  | ++ |  |  |
| **Totally found** | 21/21 | 16/21 | 15/21 | 13/21 | 11/21 |

**^a^**R03739 (consensus): **acacccatacat**; **^b^**R01817 (*aP2* gene, adipocyte P2; *S.cerevisiae*): **acacccagacmtc**; **^c^**R01331 (*rpL25* gene; *S.cerevisiae*): **aacatccgtgca**; **^d^**R01335 (*rp51A* gene; *S.cerevisiae*): **tacctccgtaca**; **^e^**R01015 (*MLP* gene, major late promoter; *S.cerevisiae*): **gatgtctgggtttt*;*** **^f^**R00576 (*alpha-2u* globulin gene; *Rattus norvegicus*): **atactgcc**; **^g^**R01435 (*TCR-delta* gene; *Homo sapiens*): **aagccctttgaa;** **^h^**R03356 (phosphoglycerate kinase 1 gene; *Mus musculus*): **aggaagttcc**; **^i^**R03887 (*snRNP E*; *Homo sapiens*): **cttccgcttccg**. Binding sites for ***Rap1p*** were considered highly variable (Idrissi and Pina, 1999; Lascaris et al., 1999). Accession numbers of known REs and their consensuses in TRANSFAC DB (Wingender et al., 2001). A "+" sign indicates the number of putative motifs found in the analyzed sequence.

These findings are supported by experimental data, showing that the high-level transcriptional activation of most ribosomal protein genes, as well as some other genes of *S.cerevisiae*, are promoted by the global DNA-binding factor Rap1p (Idrissi and Pina, 1999; Lascaris et al., 1999).


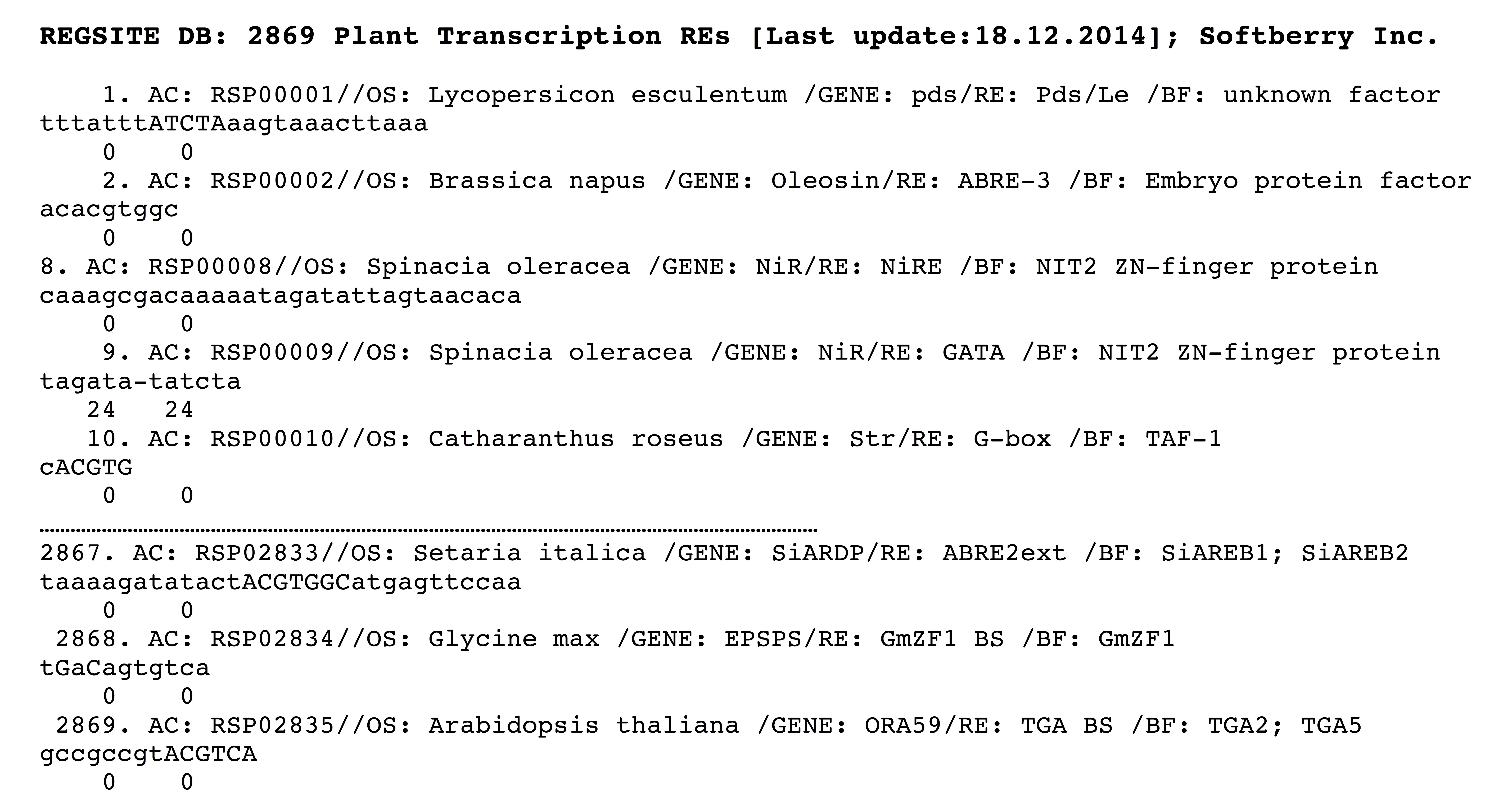


**Fig. S1.** The format of the RE dataset input file. Every RE record contains 3 lines. Line 1: accession number, AC, in the corresponding database (**ooTFD, TRANSFAC or** Regsite); organism/species, OS; gene name; RE binding factor(s), BF. Line 2 contains RE sequence (nucleotides known to be important for TF binding are given in capital). If RE consists of two boxes (composite RE), the corresponding sequences are separated by “-”. Line 3 shows minimal and maximal distances allowed (in accordance with experimental evidence) between the RE boxes.


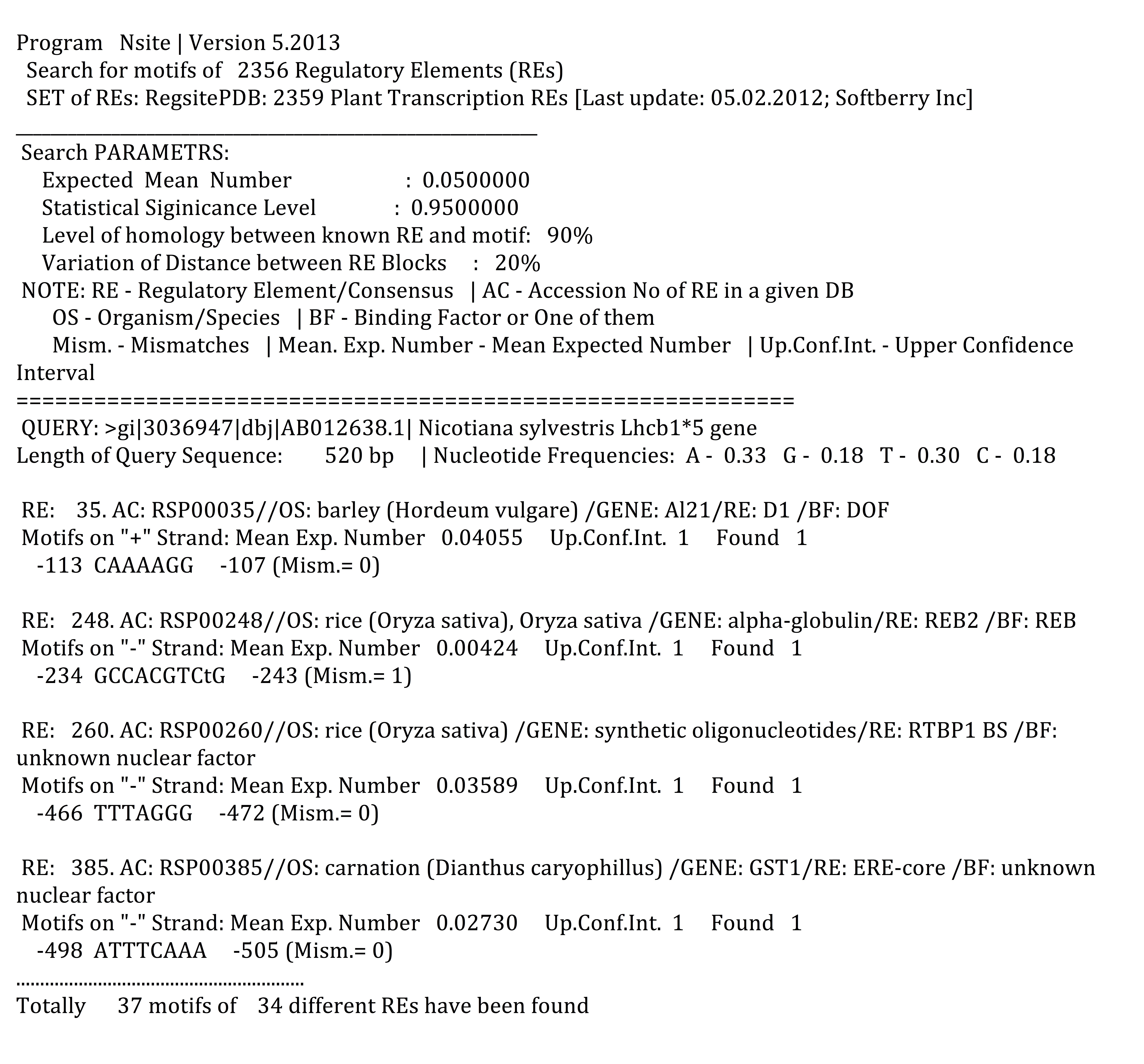


**Fig. S2.** An example of the Nsite output file. Its head contains the name of the program, search parameters, abbreviations used and a description of the query sequence. The output file consists of a description of REs identified in the analyzed sequence. A DNA strand containing a motif is denoted by a “+” or “-” sign. A line describing an identified RE motif includes: its start and end positions, number of mismatches (mismatched nucleotides are given in lower case letters) for the **1-box elements**; the start position, sequence and end position of the first box: the start position, sequence and end position of the second box; and the number of mismatches for first and second boxes separated by a “/” symbol **for composite REs**. The output file ends with a summary of detected RE motifs.





**Fig. S3. An example of the** NsiteH output file. This output is similar to the Nsite file (**Fig. 3**) but has two differences: (1) one additional parameter–a minimal level of conservation (similarity) of RE motifs (default: 80%) and (2) the description of identified motifs includes a level of conservation.

**

**

**Fig. S4.** An example of the NsiteM output file. The head of the output is similar to that for Nsite and NsiteH (see Fig. S3 and S4). The body of the output is composed of four parts: (1) the list of query sequences with given unique numbers, (2) the list of identified REs with the number of sequences where the motif is found, (3) a simple graphical view of the results and (4) detailed information on identified RE motifs (analogous to Nsite and NsiteH). In the graphical view, the first row and column indicate numbers assigned to REs and query sequences, respectively, as presented in the output. A “+” or “.” character indicate the presence or absence, respectively, of an RE motif in the query.





**Fig. S5.** The transcription regulatory motifs identified by the NsiteM program in more than 80% of the proximal promoter regions of nuclear genes encoding proteins related to Photosystem I and II in *Arabidopsis thaliana*. RSP00204 (RegSite DB): ABRE/6.2, ABI5 binding site (gacACGTggc) found in all analyzed sequences. RSP00524: E4-core, DPBF-1 and DPBF-2 binding site (ACACgtG) found in 8/10 analyzed sequences. RSP00723: G-box, HY5 binding site (cacgtggc) found in 8/10 analyzed sequences. RSP01151: Em1a, binding site for EmBP-1. Plus VP1 (GacACGTggc) found in 8/10 analyzed sequences. RSP01570: Em1a, EmBP-1 binding site (cACGTGGC) found in 8/10 analyzed sequences. RSP01816: G-box, NtbZIP binding site (gcCACGTGtc) found in 8/10 analyzed sequences. Capital letters indicate functionally important nucleotides. The corresponding output file from the NsiteM program is presented in **Supplementary File 2**.

Promoter regions of 8/10 genes contain motifs of G-box elements (RSP00723**,** RSP01816) known to be involved in the light regulation of photosynthetic genes (Siberil et al., 2001). It is known that abscisic acid (ABA) increases the net photosynthetic rate (**Hu et al., 2013 and references therein)**. Indeed, there are motifs of ABA responsive elements ABRE/6.2 (RSP00204), Em1a (RSP01151, RSP01570) and E4-core (RSP00524) in promoter regions in the majority of genes in the studied group (8/10). Moreover, the promoter locations of these motifs are largely conservative (relative to the translation start codon).

**References**

[Wingender,E et al. (2001) The TRANSFAC system on gene expression regulation. *Nucleic Acids Res*, **29**, 281-283.](http://www.ncbi.nlm.nih.gov/pubmed/?term=12.%09E.+Wingender%2CE.+et+al.+(2001)+The+TRANSFAC+system+on+gene+expression+regulation.+Nucleic+Acids+Res.%2C+29%2C+281-283)

[Idrissi, F.Z., Pina,B. (1999) Functional divergence between the half-sites of the DNA-binding sequence for the yeast transcriptional regulator Rap1p.  *Biochem J*, **341**, 477-482.](http://www.ncbi.nlm.nih.gov/pubmed/?term=Functional+divergence+between+the+half-sites+of+the+DNA-binding+sequence+for+the+yeast+transcriptional+regulator+Rap1p.++Biochem+J%2C+341%3A+477-482.)

[Lascaris, R.F. et al. (1999) DNA-binding requirements of the yeast protein Rap1p as selected in silico from ribosomal protein gene promoter sequences. *Bioinformatics*, **15**, 267-277.](http://www.ncbi.nlm.nih.gov/pubmed/?term=DNA-binding+requirements+of+the+yeast+protein+Rap1p+as+selected+in+silico+from+ribosomal+protein+gene+promoter+sequences.+Bioinformatics%2C+15%3A+267-277.)

[Hu, Y-J. et al. (2013) Effects of abscisic acid and brassinolide on photosynthetic characteristics of Leymus chinensis from Songnen Plain grassland in Northeast China. *Botanical Studies*, **54**, 42  (doi:10.1186/1999-3110-54-42)](http://www.as-botanicalstudies.com/content/54/1/42)

[Siberil, Y. et al. (2001) Plant bZIP G-box binding factors. Modular structure and activation mechanisms. *Eur J Biochem*, **268**, 5655-5666.](http://www.ncbi.nlm.nih.gov/pubmed/11722549)

[Song, Y. (2008) DNA-binding study identifies C-box and hybrid C/G-box or C/A-box motifs as high-affinity binding sites for STF1 and LONG HYPOCOTYL5 proteins. *Plant Physiol*, **146**, 1862-1877.](http://www.ncbi.nlm.nih.gov/pubmed/18287490)

[Barajas-Lopez Jde, D. et al. (2011) Circadian regulation of chloroplast f and m thioredoxins through control of the CCA1 transcription factor. *J Exp Bot*, **62**, 2039-2051.](http://www.ncbi.nlm.nih.gov/pubmed/?term=CCA1binding+Evening+Elementlight-regulated+chloroplastic+thioredoxins+m1+gene)
