# Supplementary material for: Nsite, NsiteH and NsiteM computer tools for studying transcription regulatory elements
Source: Bioinformatics. 2015 Jul 2;31(21):3544–5. doi: 10.1093/bioinformatics/btv404 (PMC4612222; doi:10.1093/bioinformatics/btv404)
Supplement: Supplementary Data [file supp_btv404_BNotes_NSITE_Sovovyev_Supplementary_2.doc]

**An output example from the program Nsite: search for statistically non-random regulatory motifs in the promoter region of the Cab-E gene encoding light-harvesting chlorophyll a/b protein in tobacco, *Nicotiana plumbaginifolia.***

Program Nsite | Version 5.2013

Search for motifs of 2869 Regulatory Elements (REs)

SET of REs: REGSITE DB: 2869 Plant Transcription REs [Last update: 18.12.2014]; Softberry Inc.

____________________________________________________________

Search PARAMETRS:

Expected Mean Number : 0.0500000

Statistical Significance Level : 0.9500000

Level of homology between known RE and motif: 90%

Variation of Distance between RE Blocks : 20%

NOTE: Mism. - Mismatches | Mean. Exp. Number - Mean Expected Number | Up.Conf.Int. - Upper Confidence Interval

============================================================

QUERY: >gi|3036947|dbj|AB012638.1| Nicotiana sylvestris Lhcb1*5 genes for light-harvesting chlorophyll a/b-binding protein, complete cds

Length of Query Sequence: 520 bp | Nucleotide Frequencies: A - 0.33 G - 0.18 T - 0.30 C - 0.18

............................................................

RE: 35. AC: RSP00035//OS: barley (Hordeum vulgare) /GENE: Al21/RE: D1 /BF: DOF

Motifs on "+" Strand: Mean Exp. Number 0.04055 Up.Conf.Int. 1 Found 1

408 CAAAAGG 414 (Mism.= 0)

............................................................

RE: 248. AC: RSP00248//OS: rice (Oryza sativa), Oryza sativa /GENE: alpha-globulin/RE: REB2 /BF: REB

Motifs on "-" Strand: Mean Exp. Number 0.00424 Up.Conf.Int. 1 Found 1

287 GCCACGTCtG 278 (Mism.= 1)

............................................................

RE: 260. AC: RSP00260//OS: rice (Oryza sativa) /GENE: synthetic oligonucleotides/RE: RTBP1 BS /BF: unknown nuclear factor

Motifs on "-" Strand: Mean Exp. Number 0.03589 Up.Conf.Int. 1 Found 1

55 TTTAGGG 49 (Mism.= 0)

............................................................

RE: 385. AC: RSP00385//OS: carnation (Dianthus caryophillus) /GENE: GST1/RE: ERE-core /BF: unknown nuclear factor

Motifs on "-" Strand: Mean Exp. Number 0.02730 Up.Conf.Int. 1 Found 1

23 ATTTCAAA 16 (Mism.= 0)

............................................................

RE: 397. AC: RSP00397//OS: tobacco (Nicotiana tabacum) /GENE: RNP2/RE: CDE /BF: unknown nuclear factor

Motifs on "-" Strand: Mean Exp. Number 0.00201 Up.Conf.Int. 1 Found 1

157 AGTGGCGG 150 (Mism.= 0)

............................................................

RE: 522. AC: RSP00522//OS: carrot (Daucus carota) /GENE: Dc3/RE: E2-core /BF: DPBF-1; DPBF-2;

Motifs on "-" Strand: Mean Exp. Number 0.01980 Up.Conf.Int. 1 Found 1

169 CCACTTG 163 (Mism.= 0)

............................................................

RE: 566. AC: RSP00566//OS: tomato (Lycopersicon esculentum), Lycopersicon esculentum /GENE: rbcS3B/RE: TATA P /BF: unknown nuclear factor

Motifs on "-" Strand: Mean Exp. Number 0.03424 Up.Conf.Int. 1 Found 2

462 CCTTGaATAT 453 (Mism.= 1)

128 tCTTGTATAT 119 (Mism.= 1)

............................................................

RE: 629. AC: RSP00629//OS: arabidopsis (Arabidopsis thaliana) /GENE: Lhcb1*3/RE: CCA1 BS2 /BF: CCA1

Motifs on "+" Strand: Mean Exp. Number 0.03485 Up.Conf.Int. 1 Found 1

356 AAAAATCT 363 (Mism.= 0)

Motifs on "-" Strand: Mean Exp. Number 0.02416 Up.Conf.Int. 1 Found 1

335 AAAAATCT 328 (Mism.= 0)

............................................................

RE: 683. AC: RSP00683//OS: arabidopsis (Arabidopsis thaliana) /GENE: Adh/RE: -190 half G-box (core) /BF: GBF3

Motifs on "+" Strand: Mean Exp. Number 0.00805 Up.Conf.Int. 1 Found 1

390 GCCAAaTGGA 399 (Mism.= 1)

............................................................

RE: 741. AC: RSP00741//OS: tobacco (Nicotiana plumbaginifolia) /GENE: Cab-E/RE: box 1 /BF: GT-1

Motifs on "+" Strand: Mean Exp. Number 0.00624 Up.Conf.Int. 1 Found 1

409 AAAAGGTTAcAA 420 (Mism.= 1)

............................................................

RE: 742. AC: RSP00742//OS: tobacco (Nicotiana plumbaginifolia) /GENE: Cab-E/RE: box 2r /BF: GT-1

Motifs on "-" Strand: Mean Exp. Number 0.00063 Up.Conf.Int. 1 Found 1

381 GCACCGTTAAAg 370 (Mism.= 1)

............................................................

RE: 819. AC: RSP00819//OS: arabidopsis (Arabidopsis thaliana) /GENE: F3H/RE: ACE-core (AtF3H) /BF: ACE-binding factor

Motifs on "-" Strand: Mean Exp. Number 0.00508 Up.Conf.Int. 1 Found 1

287 GCCACGTCTg 278 (Mism.= 1)

............................................................

RE: 836. AC: RSP00836//OS: arabidopsis (Arabidopsis thaliana) /GENE: H4A748/RE: dOCT /BF: Unknown nuclear factor

Motifs on "-" Strand: Mean Exp. Number 0.00323 Up.Conf.Int. 1 Found 1

46 CACGGATC 39 (Mism.= 0)

............................................................

RE: 852. AC: RSP00852//OS: maize (Zea mays) /GENE: H4C7/RE: dOCT /BF: Unknown nuclear factor

Motifs on "-" Strand: Mean Exp. Number 0.00323 Up.Conf.Int. 1 Found 1

46 CACGGATC 39 (Mism.= 0)

............................................................

RE: 888. AC: RSP00888//OS: arabidopsis (Arabidopsis thaliana) /GENE: AtAOX1a/RE: CCA1 motif (2) /BF: CCA1

Motifs on "+" Strand: Mean Exp. Number 0.01164 Up.Conf.Int. 1 Found 1

356 AAAAATCTA 364 (Mism.= 0)

Motifs on "-" Strand: Mean Exp. Number 0.00714 Up.Conf.Int. 1 Found 1

335 AAAAATCTA 327 (Mism.= 0)

............................................................

RE: 893. AC: RSP00893//OS: Solanum melongena /GENE: SmCP/RE: ERE 1 /BF: Dof factors

Motifs on "-" Strand: Mean Exp. Number 0.02730 Up.Conf.Int. 1 Found 1

23 ATTTCAAA 16 (Mism.= 0)

............................................................

RE: 961. AC: RSP00961//OS: barley (Hordeum vulgare) /GENE: ITR1/RE: PB1 /BF: BPBF

Motifs on "+" Strand: Mean Exp. Number 0.01198 Up.Conf.Int. 1 Found 1

406 TGCAAAAG 413 (Mism.= 0)

............................................................

RE: 1020. AC: RSP01013//OS: arabidopsis (Arabidopsis thaliana) /GENE: GapA/RE: Gap box 1 /BF: GAPF

Motifs on "+" Strand: Mean Exp. Number 0.01491 Up.Conf.Int. 1 Found 1

392 CAAATGGAaA 401 (Mism.= 1)

............................................................

RE: 1021. AC: RSP01014//OS: arabidopsis (Arabidopsis thaliana) /GENE: GapA/RE: Gap box 2 /BF: GAPF

Motifs on "-" Strand: Mean Exp. Number 0.01404 Up.Conf.Int. 1 Found 1

324 CcAATGAAGA 315 (Mism.= 1)

............................................................

RE: 1026. AC: RSP01019//OS: arabidopsis (Arabidopsis thaliana) /GENE: GapB/RE: Gap box 1 /BF: GAPF

Motifs on "-" Strand: Mean Exp. Number 0.04509 Up.Conf.Int. 1 Found 1

321 ATGAAGA 315 (Mism.= 0)

............................................................

RE: 1041. AC: RSP01034//OS: arabidopsis (Arabidopsis thaliana) /GENE: RD29B/RE: ABRE 1/2 /BF: ABI3; ABI5; AREB1

Motifs on "+" Strand: Mean Exp. Number 0.01092 Up.Conf.Int. 1 Found 1

281 ACGTGGC 287 (Mism.= 0)

............................................................

RE: 1112. AC: RSP01105//OS: spinach (Spinacia oleracea) /GENE: rps22/RE: C-rich motif /BF: unknown nuclear factor

Motifs on "+" Strand: Mean Exp. Number 0.03271 Up.Conf.Int. 1 Found 1

47 CCCCCT 52 (Mism.= 0)

............................................................

RE: 1167. AC: RSP01160//OS: tobacco (Nicotiana plumbaginifolia) /GENE: CAB/RE: G-box /BF: CG-1

Motifs on "+" Strand: Mean Exp. Number 0.00031 Up.Conf.Int. 1 Found 1

276 ATCAGACGTGGC 287 (Mism.= 0)

............................................................

RE: 1350. AC: RSP01342//OS: Arabidopsis (Arabidopsis thaliana) /GENE: cab-E/RE: GBF1 BS /BF: GBF1

Motifs on "+" Strand: Mean Exp. Number 0.00000 Up.Conf.Int. 1 Found 1

275 AATCAGACGTGGCAAA 290 (Mism.= 0)

............................................................

RE: 1539. AC: RSP01507//OS: wheat (Triticum aestivum) /GENE: H1 (TH315)/RE: Oct-p /BF:unknown transcriptin factor

Motifs on "+" Strand: Mean Exp. Number 0.00323 Up.Conf.Int. 1 Found 1

39 GATCCGTG 46 (Mism.= 0)

............................................................

RE: 1540. AC: RSP01508//OS: wheat (Triticum aestivum) /GENE: H1 (TH325)/RE: Oct-p /BF:unknown transcriptin factor

Motifs on "+" Strand: Mean Exp. Number 0.00323 Up.Conf.Int. 1 Found 1

39 GATCCGTG 46 (Mism.= 0)

............................................................

RE: 1752. AC: RSP01718//OS: Arabidopsis (Arabidopsis thaliana) /GENE: IAA3/SHY2 (At1g04240)/RE: G/A-box (1) /BF: STF1/HY5

Motifs on "+" Strand: Mean Exp. Number 0.00212 Up.Conf.Int. 1 Found 1

335 TTAaACGTATAA 346 (Mism.= 1)

............................................................

RE: 1865. AC: RSP01831//OS: Arabidopsis (Arabidopsis thaliana) /GENE: Cytc-2 (At4g10040)/RE: ACGT-motif /BF: AREB2/ABF4; GBF3; GBF-like protein; bHLH080

Motifs on "+" Strand: Mean Exp. Number 0.01923 Up.Conf.Int. 1 Found 1

337 AAACGTAT 344 (Mism.= 0)

............................................................

RE: 1977. AC: RSP01943//OS: Arabidopsis (Arabidopsis thaliana) /GENE: Synthetic oligonucleotides/RE: Hex /BF: AtbZIP1

Motifs on "+" Strand: Mean Exp. Number 0.00231 Up.Conf.Int. 1 Found 1

278 CaGACGTGGC 287 (Mism.= 1)

............................................................

RE: 2019. AC: RSP01985//OS: Arabidopsis (Arabidopsis thaliana) /GENE: NIA1/RE: Alfin1 /BF: unknown nuclear factor

Motifs on "+" Strand: Mean Exp. Number 0.01092 Up.Conf.Int. 1 Found 1

151 CGCCACT 157 (Mism.= 0)

............................................................

RE: 2064. AC: RSP02030//OS: Arabidopsis (Arabidopsis thaliana) /GENE: Synthetic oligonucleotides/RE: ERSE-I /BF: [bZIP28 + NF-YB3]

Motifs on "-" Strand: Mean Exp. Number 0.00475 Up.Conf.Int. 1 Found 1

300 CCAAT 296 --10-- 285 CACG 282 (Mism.= 0/ 0)

............................................................

RE: 2122. AC: RSP02088//OS: pea (Pisum sativum) /GENE: rbcS-E9/RE: Box III /BF: unknown nuclear factor

Motifs on "+" Strand: Mean Exp. Number 0.00879 Up.Conf.Int. 1 Found 1

93 ATCATTTTCAa 103 (Mism.= 1)

............................................................

RE: 2222. AC: RSP02188//OS: soybean (Glycine max) /GENE: CHS8/RE: Fp31/VI /BF: unknown nuclear factor

Motifs on "-" Strand: Mean Exp. Number 0.00344 Up.Conf.Int. 1 Found 1

81 ACTTGACACcC 71 (Mism.= 1)

............................................................

RE: 2322. AC: RSP02288//OS: pea (Pisum sativum) /GENE: TRX m1/RE: EE (TRX m1) /BF: CCA1

Motifs on "+" Strand: Mean Exp. Number 0.02730 Up.Conf.Int. 1 Found 1

450 TAGATATT 457 (Mism.= 0)

............................................................

RE: 2434. AC: RSP02400//OS: Arabidopsis (Arabidopsis thaliana) /GENE: COX5b/RE: distal-motif /BF: ESE1

Motifs on "-" Strand: Mean Exp. Number 0.01980 Up.Conf.Int. 1 Found 1

169 CCACTTG 163 (Mism.= 0)

............................................................

RE: 2560. AC: RSP02526//OS: rice (Oryza sativa, japonica) /GENE: OsTB1/RE: GTAC-motif 2 /BF: IPA1

Motifs on "-" Strand: Mean Exp. Number 0.01923 Up.Conf.Int. 1 Found 1

511 TTGTACTA 504 (Mism.= 0)

............................................................

RE: 2563. AC: RSP02529//OS: rice (Oryza sativa, japonica) /GENE: DEP1/RE: GTAC-motif 2 /BF: IPA1

Motifs on "-" Strand: Mean Exp. Number 0.01923 Up.Conf.Int. 1 Found 1

511 TTGTACTA 504 (Mism.= 0)

............................................................

RE: 2718. AC: RSP02684//OS: rice (Oryza sativa) /GENE: STAR1/RE: ASR5 BS /BF: ASR5

Motifs on "+" Strand: Mean Exp. Number 0.01980 Up.Conf.Int. 1 Found 1

512 AGCCCAT 518 (Mism.= 0)

............................................................

RE: 2811. AC: RSP02777//OS: Arabidopsis (Arabidopsis thaliana) /GENE: Synthetic oligonucleotides/RE: NTL8 BS /BF: NTL8

Motifs on "-" Strand: Mean Exp. Number 0.04812 Up.Conf.Int. 1 Found 1

315 ATTTCCTTCT 306 (Mism.= 1)

............................................................

RE: 2866. AC: RSP02832//OS: foxtail millet (Setaria italica) /GENE: SiARDP/RE: ABRE2 /BF: SiAREB1 ; SiAREB2

Motifs on "+" Strand: Mean Exp. Number 0.01092 Up.Conf.Int. 1 Found 1

281 ACGTGGC 287 (Mism.= 0)

............................................................

In total 43 motifs from 40 different REs have been found.

------------------------------------------------------------

**An output example from the program NsiteH: search for statistically non-random and conservative regulatory motifs in the promoter regions of the orthologous Cab-E and Lhcb1*5 genes encoding light-harvesting chlorophyll a/b protein from two species of tobacco: *Nicotiana plumbaginifolia* and *Nicotiana sylvestris*.**

Program NsiteH | Version 5.2013

Search for motifs of 2869 Regulatory Elements (REs) in a pair of Homologous Sequences

SET of REs: REGSITE DB: 2869 Plant Transcription REs [Last update: 18.12.2014]; Softberry Inc.

____________________________________________________________

Search PARAMETRS:

Expected Mean Number : 0.0500000

Statistical Significance Level : 0.9500000

Minimal Conservative Level : 80 %

Level of homology between known RE and motif: 90%

Variation of Distance between RE Blocks : 20%

NOTE: Mism. - Mismatches | Mean. Exp. Number - Mean Expected Number | Up.Conf.Int. - Upper Confidence Interval

============================================================

QUERY: >gi|19674|emb|X12512.1| Nicotiana plumbaginifolia Cab-E gene 5'-flanking region

Length of Query Sequence: 554 bp | Nucleotide Frequencies: A - 0.34 G - 0.18 T - 0.31 C - 0.16

............................................................

RE: 35. AC: RSP00035//OS: barley (Hordeum vulgare) /GENE: Al21/RE: D1 /BF: DOF

Motifs on "+" Strand: Mean Exp. Number 0.04169 Up.Conf.Int. 1 Found 1

441 CAAAAGG 447 (Mism.= 0; Cons.: 100 %)

............................................................

RE: 93. AC: RSP00093//OS: barley (Hordeum vulgare) /GENE: Amy pHV19/RE: TATCCAC box /BF: unknown nuclear factor

Motifs on "-" Strand: Mean Exp. Number 0.03964 Up.Conf.Int. 1 Found 1

288 TATCCAC 282 (Mism.= 0; Cons.: 85 %)

............................................................

RE: 248. AC: RSP00248//OS: rice (Oryza sativa), Oryza sativa /GENE: alpha-globulin/RE: REB2 /BF: REB

Motifs on "-" Strand: Mean Exp. Number 0.00345 Up.Conf.Int. 1 Found 1

320 GCCACGTCtG 311 (Mism.= 1; Cons.: 100 %)

............................................................

RE: 301. AC: RSP00301//OS: rice (Oryza sativa) (Oryza sativa) /GENE: GluB-1/RE: PROL box /BF: unknown nuclear factor

Motifs on "+" Strand: Mean Exp. Number 0.03776 Up.Conf.Int. 1 Found 1

537 TGCAAAG 543 (Mism.= 0; Cons.: 85 %)

............................................................

RE: 304. AC: RSP00304//OS: maize (Zea mays) /GENE: Synthetic oligonucleotides/RE: KN1/KIP BS /BF: KN1/KIP

Motifs on "+" Strand: Mean Exp. Number 0.01171 Up.Conf.Int. 1 Found 1

212 TGACAGGT 219 (Mism.= 0; Cons.: 87 %)

............................................................

RE: 629. AC: RSP00629//OS: arabidopsis (Arabidopsis thaliana) /GENE: Lhcb1*3/RE: CCA1 BS2 /BF: CCA1

Motifs on "+" Strand: Mean Exp. Number 0.04127 Up.Conf.Int. 1 Found 1

389 AAAAATCT 396 (Mism.= 0; Cons.: 100 %)

............................................................

RE: 683. AC: RSP00683//OS: arabidopsis (Arabidopsis thaliana) /GENE: Adh/RE: -190 half G-box (core) /BF: GBF3

Motifs on "+" Strand: Mean Exp. Number 0.00753 Up.Conf.Int. 1 Found 1

423 GCCAAaTGGA 432 (Mism.= 1; Cons.: 100 %)

............................................................

RE: 741. AC: RSP00741//OS: tobacco (Nicotiana plumbaginifolia) /GENE: Cab-E/RE: box 1 /BF: GT-1

Motifs on "+" Strand: Mean Exp. Number 0.00897 Up.Conf.Int. 1 Found 1

442 AAAAGGTTAAAA 453 (Mism.= 0; Cons.: 93 %)

............................................................

RE: 742. AC: RSP00742//OS: tobacco (Nicotiana plumbaginifolia) /GENE: Cab-E/RE: box 2r /BF: GT-1

Motifs on "-" Strand: Mean Exp. Number 0.00065 Up.Conf.Int. 1 Found 1

414 GCACCGTTAAAC 403 (Mism.= 0; Cons.: 91 %)

............................................................

RE: 819. AC: RSP00819//OS: arabidopsis (Arabidopsis thaliana) /GENE: F3H/RE: ACE-core (AtF3H) /BF: ACE-binding factor

Motifs on "-" Strand: Mean Exp. Number 0.00473 Up.Conf.Int. 1 Found 1

320 GCCACGTCTg 311 (Mism.= 1; Cons.: 100 %)

............................................................

RE: 882. AC: RSP00882//OS: maize (Zea mays) /GENE: gamma-27kDa zein/RE: Pb 3 /BF: PBF

Motifs on "+" Strand: Mean Exp. Number 0.03776 Up.Conf.Int. 1 Found 1

537 TGCAAAG 543 (Mism.= 0; Cons.: 85 %)

............................................................

RE: 889. AC: RSP00889//OS: arabidopsis (Arabidopsis thaliana) /GENE: AtAOX1a/RE: GC motif /BF: bZIP proteins

Motifs on "-" Strand: Mean Exp. Number 0.00975 Up.Conf.Int. 1 Found 1

253 CCACGCA 247 (Mism.= 0; Cons.: 85 %)

............................................................

RE: 961. AC: RSP00961//OS: barley (Hordeum vulgare) /GENE: ITR1/RE: PB1 /BF: BPBF

Motifs on "+" Strand: Mean Exp. Number 0.01299 Up.Conf.Int. 1 Found 1

439 TGCAAAAG 446 (Mism.= 0; Cons.: 100 %)

............................................................

RE: 1020. AC: RSP01013//OS: arabidopsis (Arabidopsis thaliana) /GENE: GapA/RE: Gap box 1 /BF: GAPF

Motifs on "+" Strand: Mean Exp. Number 0.01714 Up.Conf.Int. 1 Found 1

425 CAAATGGAcA 434 (Mism.= 1; Cons.: 89 %)

............................................................

RE: 1041. AC: RSP01034//OS: arabidopsis (Arabidopsis thaliana) /GENE: RD29B/RE: ABRE 1/2 /BF: ABI3; ABI5; AREB1

Motifs on "+" Strand: Mean Exp. Number 0.00929 Up.Conf.Int. 1 Found 1

314 ACGTGGC 320 (Mism.= 0; Cons.: 100 %)

............................................................

RE: 1057. AC: RSP01050//OS: barley (Hordeum vulgare) /GENE: Amy6.4/RE: GATA-core /BF: HvMCB1

Motifs on "-" Strand: Mean Exp. Number 0.03964 Up.Conf.Int. 1 Found 1

288 TATCCAC 282 (Mism.= 0; Cons.: 85 %)

............................................................

RE: 1083. AC: RSP01076//OS: Craterostigma plantagineum (Scrophulariaceae) /GENE: CpC2/RE: ABRE III /BF: CpbZIP1; CpbZIP2 (short);

Motifs on "+" Strand: Mean Exp. Number 0.00345 Up.Conf.Int. 1 Found 1

245 aATGCGTGGC 254 (Mism.= 1; Cons.: 89 %)

............................................................

RE: 1167. AC: RSP01160//OS: tobacco (Nicotiana plumbaginifolia) /GENE: CAB/RE: G-box /BF: CG-1

Motifs on "+" Strand: Mean Exp. Number 0.00026 Up.Conf.Int. 1 Found 1

309 ATCAGACGTGGC 320 (Mism.= 0; Cons.: 100 %)

............................................................

RE: 1350. AC: RSP01342//OS: Arabidopsis (Arabidopsis thaliana) /GENE: cab-E/RE: GBF1 BS /BF: GBF1

Motifs on "+" Strand: Mean Exp. Number 0.00000 Up.Conf.Int. 1 Found 1

308 AATCAGACGTGGCAAA 323 (Mism.= 0; Cons.: 100 %)

............................................................

RE: 1867. AC: RSP01833//OS: Arabidopsis (Arabidopsis thaliana) /GENE: Cytc-2 (At4g10040)/RE: Site II element /BF: AREB2/ABF4; GBF3; GBF-like protein; bHLH080;AtTCP20

Motifs on "+" Strand: Mean Exp. Number 0.04815 Up.Conf.Int. 1 Found 1

542 AGCCCA 547 (Mism.= 0; Cons.: 100 %)

............................................................

RE: 1977. AC: RSP01943//OS: Arabidopsis (Arabidopsis thaliana) /GENE: Synthetic oligonucleotides/RE: Hex /BF: AtbZIP1

Motifs on "+" Strand: Mean Exp. Number 0.00191 Up.Conf.Int. 1 Found 1

311 CaGACGTGGC 320 (Mism.= 1; Cons.: 100 %)

............................................................

RE: 2064. AC: RSP02030//OS: Arabidopsis (Arabidopsis thaliana) /GENE: Synthetic oligonucleotides/RE: ERSE-I /BF: [bZIP28 + NF-YB3]

Motifs on "-" Strand: Mean Exp. Number 0.00521 Up.Conf.Int. 1 Found 1

333 CCAAT 329 --10-- 318 CACG 315 (Mism.= 0/ 0; Cons.: 100/100 %)

............................................................

RE: 2109. AC: RSP02075//OS: Humicola gricea /GENE: pacC/RE: PacC BS1 /BF: PacC

Motifs on "+" Strand: Mean Exp. Number 0.00673 Up.Conf.Int. 1 Found 1

252 GGCCAAGA 259 (Mism.= 0; Cons.: 87 %)

............................................................

RE: 2174. AC: RSP02140//OS: maize (Zea mays) /GENE: F5H/RE: MBS-1 /BF: ZmMYB31

Motifs on "+" Strand: Mean Exp. Number 0.03763 Up.Conf.Int. 1 Found 1

460 TCCACC 465 (Mism.= 0; Cons.: 100 %)

............................................................

RE: 2216. AC: RSP02182//OS: soybean (Glycine max) /GENE: CHS8/RE: Fp23/IV /BF: unknown nuclear factor

Motifs on "+" Strand: Mean Exp. Number 0.00879 Up.Conf.Int. 1 Found 1

467 ATTGAAAAG 475 (Mism.= 0; Cons.: 88 %)

............................................................

RE: 2317. AC: RSP02283//OS: rice (Oryza sativa) /GENE: rpL32_8.1/RE: SITE II (-150) /BF: unknown nuclear factor(s)

Motifs on "+" Strand: Mean Exp. Number 0.04815 Up.Conf.Int. 1 Found 1

542 AGCCCA 547 (Mism.= 0; Cons.: 100 %)

............................................................

RE: 2372. AC: RSP02338//OS: potato (Solanum tuberosum) /GENE: StGA2ox1/RE: /BF: StBEL5

Motifs on "+" Strand: Mean Exp. Number 0.00096 Up.Conf.Int. 1 Found 1

211 TTGACAGGTCA 221 (Mism.= 1; Cons.: 90 %)

............................................................

RE: 2375. AC: RSP02341//OS: potato (Solanum tuberosum) /GENE: IPT/RE: /BF: StBEL5

Motifs on "+" Strand: Mean Exp. Number 0.00096 Up.Conf.Int. 1 Found 1

211 TTGACAGGTCA 221 (Mism.= 1; Cons.: 90 %)

............................................................

RE: 2718. AC: RSP02684//OS: rice (Oryza sativa) /GENE: STAR1/RE: ASR5 BS /BF: ASR5

Motifs on "+" Strand: Mean Exp. Number 0.01501 Up.Conf.Int. 1 Found 1

542 AGCCCAT 548 (Mism.= 0; Cons.: 100 %)

............................................................

RE: 2866. AC: RSP02832//OS: foxtail millet (Setaria italica) /GENE: SiARDP/RE: ABRE2 /BF: SiAREB1 ; SiAREB2

Motifs on "+" Strand: Mean Exp. Number 0.00929 Up.Conf.Int. 1 Found 1

314 ACGTGGC 320 (Mism.= 0; Cons.: 100 %)

............................................................

Total 49 motifs from 48 different REs have been found.

Of them: 30 motifs from 30 different REs have a Conservative Level ≥ 80 %

------------------------------------------------------------

QUERY: >gi|3036947|dbj|AB012638.1| Nicotiana sylvestris Lhcb1*5 genes for light-harvesting chlorophyll a/b-binding protein, complete cds

Length of Query Sequence: 520 bp | Nucleotide Frequencies: A - 0.33 G - 0.18 T - 0.30 C - 0.18

............................................................

RE: 35. AC: RSP00035//OS: barley (Hordeum vulgare) /GENE: Al21/RE: D1 /BF: DOF

Motifs on "+" Strand: Mean Exp. Number 0.04055 Up.Conf.Int. 1 Found 1

408 CAAAAGG 414 (Mism.= 0; Cons.: 100 %)

............................................................

RE: 248. AC: RSP00248//OS: rice (Oryza sativa), Oryza sativa /GENE: alpha-globulin/RE: REB2 /BF: REB

Motifs on "-" Strand: Mean Exp. Number 0.00424 Up.Conf.Int. 1 Found 1

287 GCCACGTCtG 278 (Mism.= 1; Cons.: 100 %)

............................................................

RE: 566. AC: RSP00566//OS: tomato (Lycopersicon esculentum), Lycopersicon esculentum /GENE: rbcS3B/RE: TATA P /BF: unknown nuclear factor

Motifs on "-" Strand: Mean Exp. Number 0.03424 Up.Conf.Int. 1 Found 1

462 CCTTGaATAT 453 (Mism.= 1; Cons.: 89 %)

............................................................

RE: 629. AC: RSP00629//OS: arabidopsis (Arabidopsis thaliana) /GENE: Lhcb1*3/RE: CCA1 BS2 /BF: CCA1

Motifs on "+" Strand: Mean Exp. Number 0.03485 Up.Conf.Int. 1 Found 1

356 AAAAATCT 363 (Mism.= 0; Cons.: 100 %)

............................................................

RE: 683. AC: RSP00683//OS: arabidopsis (Arabidopsis thaliana) /GENE: Adh/RE: -190 half G-box (core) /BF: GBF3

Motifs on "+" Strand: Mean Exp. Number 0.00805 Up.Conf.Int. 1 Found 1

390 GCCAAaTGGA 399 (Mism.= 1; Cons.: 100 %)

............................................................

RE: 741. AC: RSP00741//OS: tobacco (Nicotiana plumbaginifolia) /GENE: Cab-E/RE: box 1 /BF: GT-1

Motifs on "+" Strand: Mean Exp. Number 0.00624 Up.Conf.Int. 1 Found 1

409 AAAAGGTTAcAA 420 (Mism.= 1; Cons.: 93 %)

............................................................

RE: 742. AC: RSP00742//OS: tobacco (Nicotiana plumbaginifolia) /GENE: Cab-E/RE: box 2r /BF: GT-1

Motifs on "-" Strand: Mean Exp. Number 0.00063 Up.Conf.Int. 1 Found 1

381 GCACCGTTAAAg 370 (Mism.= 1; Cons.: 91 %)

............................................................

RE: 819. AC: RSP00819//OS: arabidopsis (Arabidopsis thaliana) /GENE: F3H/RE: ACE-core (AtF3H) /BF: ACE-binding factor

Motifs on "-" Strand: Mean Exp. Number 0.00508 Up.Conf.Int. 1 Found 1

287 GCCACGTCTg 278 (Mism.= 1; Cons.: 100 %)

............................................................

RE: 888. AC: RSP00888//OS: arabidopsis (Arabidopsis thaliana) /GENE: AtAOX1a/RE: CCA1 motif (2) /BF: CCA1

Motifs on "+" Strand: Mean Exp. Number 0.01164 Up.Conf.Int. 1 Found 1

356 AAAAATCTA 364 (Mism.= 0; Cons.: 88 %)

............................................................

RE: 961. AC: RSP00961//OS: barley (Hordeum vulgare) /GENE: ITR1/RE: PB1 /BF: BPBF

Motifs on "+" Strand: Mean Exp. Number 0.01198 Up.Conf.Int. 1 Found 1

406 TGCAAAAG 413 (Mism.= 0; Cons.: 100 %)

............................................................

RE: 1020. AC: RSP01013//OS: arabidopsis (Arabidopsis thaliana) /GENE: GapA/RE: Gap box 1 /BF: GAPF

Motifs on "+" Strand: Mean Exp. Number 0.01491 Up.Conf.Int. 1 Found 1

392 CAAATGGAaA 401 (Mism.= 1; Cons.: 89 %)

............................................................

RE: 1041. AC: RSP01034//OS: arabidopsis (Arabidopsis thaliana) /GENE: RD29B/RE: ABRE 1/2 /BF: ABI3; ABI5; AREB1

Motifs on "+" Strand: Mean Exp. Number 0.01092 Up.Conf.Int. 1 Found 1

281 ACGTGGC 287 (Mism.= 0; Cons.: 100 %)

............................................................

RE: 1167. AC: RSP01160//OS: tobacco (Nicotiana plumbaginifolia) /GENE: CAB/RE: G-box /BF: CG-1

Motifs on "+" Strand: Mean Exp. Number 0.00031 Up.Conf.Int. 1 Found 1

276 ATCAGACGTGGC 287 (Mism.= 0; Cons.: 100 %)

............................................................

RE: 1350. AC: RSP01342//OS: Arabidopsis (Arabidopsis thaliana) /GENE: cab-E/RE: GBF1 BS /BF: GBF1

Motifs on "+" Strand: Mean Exp. Number 0.00000 Up.Conf.Int. 1 Found 1

275 AATCAGACGTGGCAAA 290 (Mism.= 0; Cons.: 100 %)

............................................................

RE: 1752. AC: RSP01718//OS: Arabidopsis (Arabidopsis thaliana) /GENE: IAA3/SHY2 (At1g04240)/RE: G/A-box (1) /BF: STF1/HY5

Motifs on "+" Strand: Mean Exp. Number 0.00212 Up.Conf.Int. 1 Found 1

335 TTAaACGTATAA 346 (Mism.= 1; Cons.: 83 %)

............................................................

RE: 1977. AC: RSP01943//OS: Arabidopsis (Arabidopsis thaliana) /GENE: Synthetic oligonucleotides/RE: Hex /BF: AtbZIP1

Motifs on "+" Strand: Mean Exp. Number 0.00231 Up.Conf.Int. 1 Found 1

278 CaGACGTGGC 287 (Mism.= 1; Cons.: 100 %)

............................................................

RE: 2064. AC: RSP02030//OS: Arabidopsis (Arabidopsis thaliana) /GENE: Synthetic oligonucleotides/RE: ERSE-I /BF: [bZIP28 + NF-YB3]

Motifs on "-" Strand: Mean Exp. Number 0.00475 Up.Conf.Int. 1 Found 1

300 CCAAT 296 --10-- 285 CACG 282 (Mism.= 0/ 0; Cons.: 100/100 %)

............................................................

RE: 2322. AC: RSP02288//OS: pea (Pisum sativum) /GENE: TRX m1/RE: EE (TRX m1) /BF: CCA1

Motifs on "+" Strand: Mean Exp. Number 0.02730 Up.Conf.Int. 1 Found 1

450 TAGATATT 457 (Mism.= 0; Cons.: 87 %)

............................................................

RE: 2560. AC: RSP02526//OS: rice (Oryza sativa, japonica) /GENE: OsTB1/RE: GTAC-motif 2 /BF: IPA1

Motifs on "-" Strand: Mean Exp. Number 0.01923 Up.Conf.Int. 1 Found 1

511 TTGTACTA 504 (Mism.= 0; Cons.: 87 %)

............................................................

RE: 2563. AC: RSP02529//OS: rice (Oryza sativa, japonica) /GENE: DEP1/RE: GTAC-motif 2 /BF: IPA1

Motifs on "-" Strand: Mean Exp. Number 0.01923 Up.Conf.Int. 1 Found 1

511 TTGTACTA 504 (Mism.= 0; Cons.: 87 %)

............................................................

RE: 2718. AC: RSP02684//OS: rice (Oryza sativa) /GENE: STAR1/RE: ASR5 BS /BF: ASR5

Motifs on "+" Strand: Mean Exp. Number 0.01980 Up.Conf.Int. 1 Found 1

512 AGCCCAT 518 (Mism.= 0; Cons.: 100 %)

............................................................

RE: 2866. AC: RSP02832//OS: foxtail millet (Setaria italica) /GENE: SiARDP/RE: ABRE2 /BF: SiAREB1 ; SiAREB2

Motifs on "+" Strand: Mean Exp. Number 0.01092 Up.Conf.Int. 1 Found 1

281 ACGTGGC 287 (Mism.= 0; Cons.: 100 %)

............................................................

In total 43 motifs of 40 different REs have been found

Of them: 22 motifs of 22 different REs have a Conservative Level ≥ 80 %

------------------------------------------------------------

**An output example from the program NsiteM: search for statistically non-random motifs of 2866 known plant regulatory elements and their consensuses found in more than 80% of promoter regions of photosynthesis-related genes from *Arabidopsis thaliana* (10 genes in total).**

Program NsiteM | Version 5.2013

Search for motifs of 2869 Regulatory Elements (REs) available in a given (by USER) portion of QUERY Sequences

SET of REs: REGSITE DB: 2869 Plant Transcription REs [Last update: 18.12.2014]; Softberry Inc.

____________________________________________________________

Search PARAMETRS:

Expected Mean Number : 0.0500000

Statistical Significance Level : 0.9500000

Level of homology between known RE and motif: 90%

Variation of Distance between RE Blocks : 20%

NOTE: Mism. - Mismatches | Mean. Exp. Number - Mean Expected Number | Up.Conf.Int. - Upper Confidence Interval

============================================================

Graphic View of RE motifs found in 80% or more of analyzed sequences

MOTIFS

123456789

________________

SEQs

1| .++..+.+.

2| ++.++++++

3| ++.++++++

4| .++..+.+.

5| +++++++++

6| +++++++++

7| +++++++++

8| +++++.+++

9| +++++.+++

10| +++++++++

Motifs of RE No 1 | NAME: 175. AC: RSP00175//OS: arabidopsis (Arabidopsis thaliana) /GENE: synthetic oligonucleotides/RE: ABRE /BF: ABFs | SEQ: cacgtggc

Found in the following 8 ( 80.0%) out of 10 analyzed sequences:

2 3 5 6 7 8 9 10

Motifs of RE No 2 | NAME: 204. AC: RSP00204//OS: arabidopsis (Arabidopsis thaliana) /GENE: AtEm6/RE: ABRE/6.2 /BF: ABI5 | SEQ: gacACGTggc

Found in the following 10 ( 100.0%) out of 10 analyzed sequences:

1 2 3 4 5 6 7 8 9 10

Motifs of RE No 3 | NAME: 524. AC: RSP00524//OS: carrot (Daucus carota) /GENE: Dc3/RE: E4-core /BF: DPBF-1; DPBF-2; | SEQ: ACACgtG

Found in the following 8 ( 80.0%) out of 10 analyzed sequences:

1 4 5 6 7 8 9 10

Motifs of RE No 4 | NAME: 723. AC: RSP00723//OS: tobacco (Nicotiana plumbaginifolia) /GENE: rbcS 8B/RE: G-box /BF: HY5 | SEQ: cacgtggc

Found in the following 8 ( 80.0%) out of 10 analyzed sequences:

2 3 5 6 7 8 9 10

Motifs of RE No 5 | NAME: 1041. AC: RSP01034//OS: arabidopsis (Arabidopsis thaliana) /GENE: RD29B/RE: ABRE 1/2 /BF: ABI3; ABI5; AREB1 | SEQ: ACGTGgC

Found in the following 8 ( 80.0%) out of 10 analyzed sequences:

2 3 5 6 7 8 9 10

Motifs of RE No 6 | NAME: 1159. AC: RSP01151//OS: maize (Zea mays) /GENE: Em/RE: Em1a /BF: EmBP-1 (+VP1) | SEQ: GacACGTggc

Found in the following 8 ( 80.0%) out of 10 analyzed sequences:

1 2 3 4 5 6 7 10

Motifs of RE No 7 | NAME: 1602. AC: RSP01570//OS: rice (Oryza sativa, indica) /GENE: Synthetic oligonucleotides/RE: Em1a /BF: OSBZ | SEQ: cACGTGGC

Found in the following 8 ( 80.0%) out of 10 analyzed sequences:

2 3 5 6 7 8 9 10

Motifs of RE No 8 | NAME: 1850. AC: RSP01816//OS: tobacco (Nicotiana tabacum) /GENE: PNZIP/RE: G-box /BF: NtbZIP | SEQ: gcCACGTGtc

Found in the following 10 ( 100.0%) out of 10 analyzed sequences:

1 2 3 4 5 6 7 8 9 10

Motifs of RE No 9 | NAME: 2866. AC: RSP02832//OS: foxtail millet (Setaria italica) /GENE: SiARDP/RE: ABRE2 /BF: SiAREB1 ; SiAREB2 | SEQ: ACGTGGC

Found in the following 8 ( 80.0%) out of 10 analyzed sequences:

2 3 5 6 7 8 9 10

============================================================

LIST of sequences presented in the graphic view of the results.

1. EXAMPLE Group (10 genes) | Arabidopsis, Chr 1: GENE: F21B7.21 [At1g03600] |898875..899654 |SUPPORT | 1 exon(s) |PROD: photosystem II protein family |5"-UTR: 40 | -500: +1 | +1 ... CDS start

2. EXAMPLE Group (10 genes) | Arabidopsis, Chr 1: GENE: F4H5.23 [At1g06680] |2047878..2049417 |SUPPORT | 4 exon(s) |PROD: photosystem II oxygen-evolving complex 23 (OEC23) |5"-UTR: 61 | -500: +1 |

3. EXAMPLE Group (10 genes) | Arabidopsis, Chr 1: GENE: F9I5.11 [At1g52230] |19520367..19521199 |SUPPORT | 3 exon(s) |PROD: photosystem I subunit VI precursor |5"-UTR: 66 | -500: +1 | +1 ... CDS st

4. EXAMPLE Group (10 genes) | Arabidopsis, Chr 2: GENE: T6B20.8 [At2g30570] |complement(13025520..13027271) |SUPPORT | 2 exon(s) |PROD: photosystem II reaction center 6.1KD protein |5"-UTR: 141 | -50

5. EXAMPLE Group (10 genes) | Arabidopsis, Chr 3: GENE: MSL1.18 [At3g16140] |complement(5468517..5469482) |SUPPORT | 3 exon(s) |PROD: photosystem I subunit VI precursor |5"-UTR: 61 | -500: +1 | +1

6. EXAMPLE Group (10 genes) | Arabidopsis, Chr 3: GENE: MSA6.6 [At3g21055] |complement(7376643..7377192) |SUPPORT | 1 exon(s) |PROD: photosystem II 5 kD protein precursor |5"-UTR: 114 | -500: +1 | +

7. EXAMPLE Group (10 genes) | Arabidopsis, Chr 3: GENE: F18B3.100 [At3g50820] |complement(18901857..18903407) |SUPPORT | 3 exon(s) |PROD: photosystem II oxygen-evolving complex 33 (OEC33) |5"-UTR: 11

8. EXAMPLE Group (10 genes) | Arabidopsis, Chr 4: GENE: T5F17.110 [At4g28660] |14149922..14151103 |SUPPORT | 2 exon(s) |PROD: photosystem II protein W - like |5"-UTR: 92 | -500: +1 | +1 ... CDS sta

9. EXAMPLE Group (10 genes) | Arabidopsis, Chr 4: GENE: F16A16.140 [At4g28750] |complement(14202779..14203961) |SUPPORT | 3 exon(s) |PROD: photosystem I subunit PSI-E - like protein |5"-UTR: 67 | -5

10. EXAMPLE Group (10 genes) | Arabidopsis, Chr 5: GENE: K1F13.25 [At5g66570] |26585884..26587504 |SUPPORT | 3 exon(s) |PROD: photosystem II oxygen-evolving complex 33 (OEC33) |5"-UTR: 86 | -500: +1

============================================================

RE motifs were found in 80% or more of analyzed sequences.

............................................................

1. QUERY: > EXAMPLE Group (10 genes) | Arabidopsis, Chr 1: GENE: F21B7.21 [At1g03600] |898875..899654 |SUPPORT | 1 exon(s) |PROD: photosystem II protein family |5"-UTR: 40 | -500: +1 | +1 ... CDS start

Length of Query Sequence: 500 bp | Nucleotide Frequencies: A - 0.37 G - 0.15 T - 0.27 C - 0.21

............................................................

RE: 204. AC: RSP00204//OS: arabidopsis (Arabidopsis thaliana) /GENE: AtEm6/RE: ABRE/6.2 /BF: ABI5

Found in 10 (100.00 %) SEQs (out of 10)

Motifs on "+" Strand: Mean Exp. Number 0.00232 Found 1

393 GACACGTGtC 402 (Mism.= 1)

Motifs on "-" Strand: Mean Exp. Number 0.00223 Found 1

402 GACACGTGtC 393 (Mism.= 1)

............................................................

RE: 524. AC: RSP00524//OS: carrot (Daucus carota) /GENE: Dc3/RE: E4-core /BF: DPBF-1; DPBF-2;

Found in 8 ( 80.00 %) SEQs (out of 10)

Motifs on "+" Strand: Mean Exp. Number 0.01810 Found 1

394 ACACGTG 400 (Mism.= 0)

Motifs on "-" Strand: Mean Exp. Number 0.01313 Found 2

401 ACACGTG 395 (Mism.= 0)

155 ACACGTG 149 (Mism.= 0)

............................................................

RE: 1159. AC: RSP01151//OS: maize (Zea mays) /GENE: Em/RE: Em1a /BF: EmBP-1 (+VP1)

Found in 8 ( 80.00 %) SEQs (out of 10)

Motifs on "+" Strand: Mean Exp. Number 0.00185 Found 1

393 GACACGTGtC 402 (Mism.= 1)

Motifs on "-" Strand: Mean Exp. Number 0.00190 Found 1

402 GACACGTGTC 393 (Mism.= 1)

............................................................

RE: 1850. AC: RSP01816//OS: tobacco (Nicotiana tabacum) /GENE: PNZIP/RE: G-box /BF: NtbZIP

Found in 10 (100.00 %) SEQs (out of 10)

Motifs on "+" Strand: Mean Exp. Number 0.00143 Found 1

393 GaCACGTGTC 402 (Mism.= 1)

Motifs on "-" Strand: Mean Exp. Number 0.00152 Found 1

402 GaCACGTGTC 393 (Mism.= 1)

............................................................

In total 9 motifs from 4 different REs have been found.

------------------------------------------------------------

2. QUERY: > EXAMPLE Group (10 genes) | Arabidopsis, Chr 1: GENE: F4H5.23 [At1g06680] |2047878..2049417 |SUPPORT | 4 exon(s) |PROD: photosystem II oxygen-evolving complex 23 (OEC23) |5"-UTR: 61 | -500: +1 |

Length of Query Sequence: 500 bp | Nucleotide Frequencies: A - 0.37 G - 0.17 T - 0.26 C - 0.19

............................................................

RE: 175. AC: RSP00175//OS: arabidopsis (Arabidopsis thaliana) /GENE: synthetic oligonucleotides/RE: ABRE /BF: ABFs

Found in 8 ( 80.00 %) SEQs (out of 10)

Motifs on "+" Strand: Mean Exp. Number 0.00175 Found 1

301 CACGTGGC 308 (Mism.= 0)

Motifs on "-" Strand: Mean Exp. Number 0.00175 Found 1

306 CACGTGGC 299 (Mism.= 0)

............................................................

RE: 204. AC: RSP00204//OS: arabidopsis (Arabidopsis thaliana) /GENE: AtEm6/RE: ABRE/6.2 /BF: ABI5

Found in 10 (100.00 %) SEQs (out of 10)

Motifs on "+" Strand: Mean Exp. Number 0.00288 Found 1

299 GcCACGTGGC 308 (Mism.= 1)

Motifs on "-" Strand: Mean Exp. Number 0.00226 Found 1

308 GcCACGTGGC 299 (Mism.= 1)

............................................................

RE: 723. AC: RSP00723//OS: tobacco (Nicotiana plumbaginifolia) /GENE: rbcS 8B/RE: G-box /BF: HY5

Found in 8 ( 80.00 %) SEQs (out of 10)

Motifs on "+" Strand: Mean Exp. Number 0.00175 Found 1

301 CACGTGGC 308 (Mism.= 0)

Motifs on "-" Strand: Mean Exp. Number 0.00175 Found 1

306 CACGTGGC 299 (Mism.= 0)

............................................................

RE: 1041. AC: RSP01034//OS: arabidopsis (Arabidopsis thaliana) /GENE: RD29B/RE: ABRE 1/2 /BF: ABI3; ABI5; AREB1

Found in 8 ( 80.00 %) SEQs (out of 10)

Motifs on "+" Strand: Mean Exp. Number 0.00921 Found 1

302 ACGTGGC 308 (Mism.= 0)

Motifs on "-" Strand: Mean Exp. Number 0.01005 Found 1

305 ACGTGGC 299 (Mism.= 0)

............................................................

RE: 1159. AC: RSP01151//OS: maize (Zea mays) /GENE: Em/RE: Em1a /BF: EmBP-1 (+VP1)

Found in 8 ( 80.00 %) SEQs (out of 10)

Motifs on "+" Strand: Mean Exp. Number 0.00234 Found 1

299 GCCACGTGGC 308 (Mism.= 1)

Motifs on "-" Strand: Mean Exp. Number 0.00189 Found 1

308 GCCACGTGGC 299 (Mism.= 1)

............................................................

RE: 1602. AC: RSP01570//OS: rice (Oryza sativa, indica) /GENE: Synthetic oligonucleotides/RE: Em1a /BF: OSBZ

Found in 8 ( 80.00 %) SEQs (out of 10)

Motifs on "+" Strand: Mean Exp. Number 0.00175 Found 1

301 CACGTGGC 308 (Mism.= 0)

Motifs on "-" Strand: Mean Exp. Number 0.00175 Found 1

306 CACGTGGC 299 (Mism.= 0)

............................................................

RE: 1850. AC: RSP01816//OS: tobacco (Nicotiana tabacum) /GENE: PNZIP/RE: G-box /BF: NtbZIP

Found in 10 (100.00 %) SEQs (out of 10)

Motifs on "+" Strand: Mean Exp. Number 0.00148 Found 1

299 GCCACGTGgC 308 (Mism.= 1)

Motifs on "-" Strand: Mean Exp. Number 0.00186 Found 1

308 GCCACGTGgC 299 (Mism.= 1)

............................................................

RE: 2866. AC: RSP02832//OS: foxtail millet (Setaria italica) /GENE: SiARDP/RE: ABRE2 /BF: SiAREB1 ; SiAREB2

Found in 8 ( 80.00 %) SEQs (out of 10)

Motifs on "+" Strand: Mean Exp. Number 0.00921 Found 1

302 ACGTGGC 308 (Mism.= 0)

Motifs on "-" Strand: Mean Exp. Number 0.01005 Found 1

305 ACGTGGC 299 (Mism.= 0)

............................................................

In total 16 motifs from 8 different REs have been found.

------------------------------------------------------------

3. QUERY: > EXAMPLE Group (10 genes) | Arabidopsis, Chr 1: GENE: F9I5.11 [At1g52230] |19520367..19521199 |SUPPORT | 3 exon(s) |PROD: photosystem I subunit VI precursor |5"-UTR: 66 | -500: +1 | +1 ... CDS st

Length of Query Sequence: 500 bp | Nucleotide Frequencies: A - 0.37 G - 0.20 T - 0.27 C - 0.17

............................................................

RE: 175. AC: RSP00175//OS: arabidopsis (Arabidopsis thaliana) /GENE: synthetic oligonucleotides/RE: ABRE /BF: ABFs

Found in 8 ( 80.00 %) SEQs (out of 10)

Motifs on "+" Strand: Mean Exp. Number 0.00174 Found 2

329 CACGTGGC 336 (Mism.= 0)

384 CACGTGGC 391 (Mism.= 0)

Motifs on "-" Strand: Mean Exp. Number 0.00174 Found 1

389 CACGTGGC 382 (Mism.= 0)

............................................................

RE: 204. AC: RSP00204//OS: arabidopsis (Arabidopsis thaliana) /GENE: AtEm6/RE: ABRE/6.2 /BF: ABI5

Found in 10 (100.00 %) SEQs (out of 10)

Motifs on "+" Strand: Mean Exp. Number 0.00310 Found 1

382 GcCACGTGGC 391 (Mism.= 1)

Motifs on "-" Strand: Mean Exp. Number 0.00210 Found 1

391 GcCACGTGGC 382 (Mism.= 1)

............................................................

RE: 723. AC: RSP00723//OS: tobacco (Nicotiana plumbaginifolia) /GENE: rbcS 8B/RE: G-box /BF: HY5

Found in 8 ( 80.00 %) SEQs (out of 10)

Motifs on "+" Strand: Mean Exp. Number 0.00174 Found 2

329 CACGTGGC 336 (Mism.= 0)

384 CACGTGGC 391 (Mism.= 0)

Motifs on "-" Strand: Mean Exp. Number 0.00174 Found 1

389 CACGTGGC 382 (Mism.= 0)

............................................................

RE: 1041. AC: RSP01034//OS: arabidopsis (Arabidopsis thaliana) /GENE: RD29B/RE: ABRE 1/2 /BF: ABI3; ABI5; AREB1

Found in 8 ( 80.00 %) SEQs (out of 10)

Motifs on "+" Strand: Mean Exp. Number 0.01037 Found 2

330 ACGTGGC 336 (Mism.= 0)

385 ACGTGGC 391 (Mism.= 0)

Motifs on "-" Strand: Mean Exp. Number 0.00889 Found 1

388 ACGTGGC 382 (Mism.= 0)

............................................................

RE: 1159. AC: RSP01151//OS: maize (Zea mays) /GENE: Em/RE: Em1a /BF: EmBP-1 (+VP1)

Found in 8 ( 80.00 %) SEQs (out of 10)

Motifs on "+" Strand: Mean Exp. Number 0.00259 Found 1

382 GcCACGTGGC 391 (Mism.= 1)

Motifs on "-" Strand: Mean Exp. Number 0.00171 Found 1

391 GCCACGTGGC 382 (Mism.= 1)

............................................................

RE: 1602. AC: RSP01570//OS: rice (Oryza sativa, indica) /GENE: Synthetic oligonucleotides/RE: Em1a /BF: OSBZ

Found in 8 ( 80.00 %) SEQs (out of 10)

Motifs on "+" Strand: Mean Exp. Number 0.00174 Found 2

329 CACGTGGC 336 (Mism.= 0)

384 CACGTGGC 391 (Mism.= 0)

Motifs on "-" Strand: Mean Exp. Number 0.00174 Found 1

389 CACGTGGC 382 (Mism.= 0)

............................................................

RE: 1850. AC: RSP01816//OS: tobacco (Nicotiana tabacum) /GENE: PNZIP/RE: G-box /BF: NtbZIP

Found in 10 (100.00 %) SEQs (out of 10)

Motifs on "+" Strand: Mean Exp. Number 0.00139 Found 1

382 GCCACGTGgC 391 (Mism.= 1)

Motifs on "-" Strand: Mean Exp. Number 0.00197 Found 1

391 GCCACGTGgC 382 (Mism.= 1)

............................................................

RE: 2866. AC: RSP02832//OS: foxtail millet (Setaria italica) /GENE: SiARDP/RE: ABRE2 /BF: SiAREB1 ; SiAREB2

Found in 8 ( 80.00 %) SEQs (out of 10)

Motifs on "+" Strand: Mean Exp. Number 0.01037 Found 2

330 ACGTGGC 336 (Mism.= 0)

385 ACGTGGC 391 (Mism.= 0)

Motifs on "-" Strand: Mean Exp. Number 0.00889 Found 1

388 ACGTGGC 382 (Mism.= 0)

............................................................

In total 21 motifs from 8 different REs have been found.

------------------------------------------------------------

4. QUERY: > EXAMPLE Group (10 genes) | Arabidopsis, Chr 2: GENE: T6B20.8 [At2g30570] |complement(13025520..13027271) |SUPPORT | 2 exon(s) |PROD: photosystem II reaction center 6.1KD protein |5"-UTR: 141 | -50

Length of Query Sequence: 500 bp | Nucleotide Frequencies: A - 0.34 G - 0.20 T - 0.31 C - 0.14

............................................................

RE: 204. AC: RSP00204//OS: arabidopsis (Arabidopsis thaliana) /GENE: AtEm6/RE: ABRE/6.2 /BF: ABI5

Found in 10 (100.00 %) SEQs (out of 10)

Motifs on "+" Strand: Mean Exp. Number 0.00238 Found 1

275 GACACGTGGa 284 (Mism.= 1)

............................................................

RE: 524. AC: RSP00524//OS: carrot (Daucus carota) /GENE: Dc3/RE: E4-core /BF: DPBF-1; DPBF-2;

Found in 8 ( 80.00 %) SEQs (out of 10)

Motifs on "+" Strand: Mean Exp. Number 0.01525 Found 1

276 ACACGTG 282 (Mism.= 0)

............................................................

RE: 1159. AC: RSP01151//OS: maize (Zea mays) /GENE: Em/RE: Em1a /BF: EmBP-1 (+VP1)

Found in 8 ( 80.00 %) SEQs (out of 10)

Motifs on "+" Strand: Mean Exp. Number 0.00202 Found 1

275 GACACGTGGA 284 (Mism.= 1)

............................................................

RE: 1850. AC: RSP01816//OS: tobacco (Nicotiana tabacum) /GENE: PNZIP/RE: G-box /BF: NtbZIP

Found in 10 (100.00 %) SEQs (out of 10)

Motifs on "-" Strand: Mean Exp. Number 0.00149 Found 1

284 tCCACGTGTC 275 (Mism.= 1)

............................................................

In total 4 motifs from 4 different REs have been found.

------------------------------------------------------------

5. QUERY: > EXAMPLE Group (10 genes) | Arabidopsis, Chr 3: GENE: MSL1.18 [At3g16140] |complement(5468517..5469482) |SUPPORT | 3 exon(s) |PROD: photosystem I subunit VI precursor |5"-UTR: 61 | -500: +1 | +1

Length of Query Sequence: 500 bp | Nucleotide Frequencies: A - 0.36 G - 0.17 T - 0.28 C - 0.19

............................................................

RE: 175. AC: RSP00175//OS: arabidopsis (Arabidopsis thaliana) /GENE: synthetic oligonucleotides/RE: ABRE /BF: ABFs

Found in 8 ( 80.00 %) SEQs (out of 10)

Motifs on "+" Strand: Mean Exp. Number 0.00178 Found 1

339 CACGTGGC 346 (Mism.= 0)

Motifs on "-" Strand: Mean Exp. Number 0.00178 Found 1

397 CACGTGGC 390 (Mism.= 0)

............................................................

RE: 204. AC: RSP00204//OS: arabidopsis (Arabidopsis thaliana) /GENE: AtEm6/RE: ABRE/6.2 /BF: ABI5

Found in 10 (100.00 %) SEQs (out of 10)

Motifs on "-" Strand: Mean Exp. Number 0.00243 Found 1

399 GACACGTGGC 390 (Mism.= 0)

............................................................

RE: 524. AC: RSP00524//OS: carrot (Daucus carota) /GENE: Dc3/RE: E4-core /BF: DPBF-1; DPBF-2;

Found in 8 ( 80.00 %) SEQs (out of 10)

Motifs on "-" Strand: Mean Exp. Number 0.01507 Found 1

398 ACACGTG 392 (Mism.= 0)

............................................................

RE: 723. AC: RSP00723//OS: tobacco (Nicotiana plumbaginifolia) /GENE: rbcS 8B/RE: G-box /BF: HY5

Found in 8 ( 80.00 %) SEQs (out of 10)

Motifs on "+" Strand: Mean Exp. Number 0.00178 Found 1

339 CACGTGGC 346 (Mism.= 0)

Motifs on "-" Strand: Mean Exp. Number 0.00178 Found 1

397 CACGTGGC 390 (Mism.= 0)

............................................................

RE: 1041. AC: RSP01034//OS: arabidopsis (Arabidopsis thaliana) /GENE: RD29B/RE: ABRE 1/2 /BF: ABI3; ABI5; AREB1

Found in 8 ( 80.00 %) SEQs (out of 10)

Motifs on "+" Strand: Mean Exp. Number 0.00936 Found 1

340 ACGTGGC 346 (Mism.= 0)

Motifs on "-" Strand: Mean Exp. Number 0.01023 Found 1

396 ACGTGGC 390 (Mism.= 0)

............................................................

RE: 1159. AC: RSP01151//OS: maize (Zea mays) /GENE: Em/RE: Em1a /BF: EmBP-1 (+VP1)

Found in 8 ( 80.00 %) SEQs (out of 10)

Motifs on "-" Strand: Mean Exp. Number 0.00203 Found 1

399 GACACGTGGC 390 (Mism.= 0)

............................................................

RE: 1602. AC: RSP01570//OS: rice (Oryza sativa, indica) /GENE: Synthetic oligonucleotides/RE: Em1a /BF: OSBZ

Found in 8 ( 80.00 %) SEQs (out of 10)

Motifs on "+" Strand: Mean Exp. Number 0.00178 Found 1

339 CACGTGGC 346 (Mism.= 0)

Motifs on "-" Strand: Mean Exp. Number 0.00178 Found 1

397 CACGTGGC 390 (Mism.= 0)

............................................................

RE: 1850. AC: RSP01816//OS: tobacco (Nicotiana tabacum) /GENE: PNZIP/RE: G-box /BF: NtbZIP

Found in 10 (100.00 %) SEQs (out of 10)

Motifs on "+" Strand: Mean Exp. Number 0.00158 Found 1

390 GCCACGTGTC 399 (Mism.= 0)

............................................................

RE: 2866. AC: RSP02832//OS: foxtail millet (Setaria italica) /GENE: SiARDP/RE: ABRE2 /BF: SiAREB1 ; SiAREB2

Found in 8 ( 80.00 %) SEQs (out of 10)

Motifs on "+" Strand: Mean Exp. Number 0.00936 Found 1

340 ACGTGGC 346 (Mism.= 0)

Motifs on "-" Strand: Mean Exp. Number 0.01023 Found 1

396 ACGTGGC 390 (Mism.= 0)

............................................................

In total 14 motifs from 9 different REs have been found.

------------------------------------------------------------

6. QUERY: > EXAMPLE Group (10 genes) | Arabidopsis, Chr 3: GENE: MSA6.6 [At3g21055] |complement(7376643..7377192) |SUPPORT | 1 exon(s) |PROD: photosystem II 5 kD protein precursor |5"-UTR: 114 | -500: +1 | +

Length of Query Sequence: 500 bp | Nucleotide Frequencies: A - 0.35 G - 0.20 T - 0.26 C - 0.19

............................................................

RE: 175. AC: RSP00175//OS: arabidopsis (Arabidopsis thaliana) /GENE: synthetic oligonucleotides/RE: ABRE /BF: ABFs

Found in 8 ( 80.00 %) SEQs (out of 10)

Motifs on "+" Strand: Mean Exp. Number 0.00240 Found 1

333 CACGTGGC 340 (Mism.= 0)

............................................................

RE: 204. AC: RSP00204//OS: arabidopsis (Arabidopsis thaliana) /GENE: AtEm6/RE: ABRE/6.2 /BF: ABI5

Found in 10 (100.00 %) SEQs (out of 10)

Motifs on "+" Strand: Mean Exp. Number 0.00392 Found 1

331 GACACGTGGC 340 (Mism.= 0)

............................................................

RE: 524. AC: RSP00524//OS: carrot (Daucus carota) /GENE: Dc3/RE: E4-core /BF: DPBF-1; DPBF-2;

Found in 8 ( 80.00 %) SEQs (out of 10)

Motifs on "+" Strand: Mean Exp. Number 0.02252 Found 1

332 ACACGTG 338 (Mism.= 0)

............................................................

RE: 723. AC: RSP00723//OS: tobacco (Nicotiana plumbaginifolia) /GENE: rbcS 8B/RE: G-box /BF: HY5

Found in 8 ( 80.00 %) SEQs (out of 10)

Motifs on "+" Strand: Mean Exp. Number 0.00240 Found 1

333 CACGTGGC 340 (Mism.= 0)

............................................................

RE: 1041. AC: RSP01034//OS: arabidopsis (Arabidopsis thaliana) /GENE: RD29B/RE: ABRE 1/2 /BF: ABI3; ABI5; AREB1

Found in 8 ( 80.00 %) SEQs (out of 10)

Motifs on "+" Strand: Mean Exp. Number 0.01267 Found 1

334 ACGTGGC 340 (Mism.= 0)

............................................................

RE: 1159. AC: RSP01151//OS: maize (Zea mays) /GENE: Em/RE: Em1a /BF: EmBP-1 (+VP1)

Found in 8 ( 80.00 %) SEQs (out of 10)

Motifs on "+" Strand: Mean Exp. Number 0.00325 Found 1

331 GACACGTGGC 340 (Mism.= 0)

............................................................

RE: 1602. AC: RSP01570//OS: rice (Oryza sativa, indica) /GENE: Synthetic oligonucleotides/RE: Em1a /BF: OSBZ

Found in 8 ( 80.00 %) SEQs (out of 10)

Motifs on "+" Strand: Mean Exp. Number 0.00240 Found 1

333 CACGTGGC 340 (Mism.= 0)

............................................................

RE: 1850. AC: RSP01816//OS: tobacco (Nicotiana tabacum) /GENE: PNZIP/RE: G-box /BF: NtbZIP

Found in 10 (100.00 %) SEQs (out of 10)

Motifs on "-" Strand: Mean Exp. Number 0.00254 Found 1

340 GCCACGTGTC 331 (Mism.= 0)

............................................................

RE: 2866. AC: RSP02832//OS: foxtail millet (Setaria italica) /GENE: SiARDP/RE: ABRE2 /BF: SiAREB1 ; SiAREB2

Found in 8 ( 80.00 %) SEQs (out of 10)

Motifs on "+" Strand: Mean Exp. Number 0.01267 Found 1

334 ACGTGGC 340 (Mism.= 0)

............................................................

In total 9 motifs from 9 different REs have been found.

------------------------------------------------------------

7. QUERY: > EXAMPLE Group (10 genes) | Arabidopsis, Chr 3: GENE: F18B3.100 [At3g50820] |complement(18901857..18903407) |SUPPORT | 3 exon(s) |PROD: photosystem II oxygen-evolving complex 33 (OEC33) |5"-UTR: 11

Length of Query Sequence: 500 bp | Nucleotide Frequencies: A - 0.33 G - 0.12 T - 0.35 C - 0.20

............................................................

RE: 175. AC: RSP00175//OS: arabidopsis (Arabidopsis thaliana) /GENE: synthetic oligonucleotides/RE: ABRE /BF: ABFs

Found in 8 ( 80.00 %) SEQs (out of 10)

Motifs on "+" Strand: Mean Exp. Number 0.00073 Found 1

336 CACGTGGC 343 (Mism.= 0)

............................................................

RE: 204. AC: RSP00204//OS: arabidopsis (Arabidopsis thaliana) /GENE: AtEm6/RE: ABRE/6.2 /BF: ABI5

Found in 10 (100.00 %) SEQs (out of 10)

Motifs on "+" Strand: Mean Exp. Number 0.00096 Found 1

334 GACACGTGGC 343 (Mism.= 0)

............................................................

RE: 524. AC: RSP00524//OS: carrot (Daucus carota) /GENE: Dc3/RE: E4-core /BF: DPBF-1; DPBF-2;

Found in 8 ( 80.00 %) SEQs (out of 10)

Motifs on "+" Strand: Mean Exp. Number 0.01040 Found 1

335 ACACGTG 341 (Mism.= 0)

............................................................

RE: 723. AC: RSP00723//OS: tobacco (Nicotiana plumbaginifolia) /GENE: rbcS 8B/RE: G-box /BF: HY5

Found in 8 ( 80.00 %) SEQs (out of 10)

Motifs on "+" Strand: Mean Exp. Number 0.00073 Found 1

336 CACGTGGC 343 (Mism.= 0)

............................................................

RE: 1041. AC: RSP01034//OS: arabidopsis (Arabidopsis thaliana) /GENE: RD29B/RE: ABRE 1/2 /BF: ABI3; ABI5; AREB1

Found in 8 ( 80.00 %) SEQs (out of 10)

Motifs on "+" Strand: Mean Exp. Number 0.00372 Found 1

337 ACGTGGC 343 (Mism.= 0)

............................................................

RE: 1159. AC: RSP01151//OS: maize (Zea mays) /GENE: Em/RE: Em1a /BF: EmBP-1 (+VP1)

Found in 8 ( 80.00 %) SEQs (out of 10)

Motifs on "+" Strand: Mean Exp. Number 0.00074 Found 1

334 GACACGTGGC 343 (Mism.= 0)

............................................................

RE: 1602. AC: RSP01570//OS: rice (Oryza sativa, indica) /GENE: Synthetic oligonucleotides/RE: Em1a /BF: OSBZ

Found in 8 ( 80.00 %) SEQs (out of 10)

Motifs on "+" Strand: Mean Exp. Number 0.00073 Found 1

336 CACGTGGC 343 (Mism.= 0)

............................................................

RE: 1850. AC: RSP01816//OS: tobacco (Nicotiana tabacum) /GENE: PNZIP/RE: G-box /BF: NtbZIP

Found in 10 (100.00 %) SEQs (out of 10)

Motifs on "-" Strand: Mean Exp. Number 0.00063 Found 1

343 GCCACGTGTC 334 (Mism.= 0)

............................................................

RE: 2866. AC: RSP02832//OS: foxtail millet (Setaria italica) /GENE: SiARDP/RE: ABRE2 /BF: SiAREB1 ; SiAREB2

Found in 8 ( 80.00 %) SEQs (out of 10)

Motifs on "+" Strand: Mean Exp. Number 0.00372 Found 1

337 ACGTGGC 343 (Mism.= 0)

............................................................

In total 9 motifs from 9 different REs have been found.

------------------------------------------------------------

8. QUERY: > EXAMPLE Group (10 genes) | Arabidopsis, Chr 4: GENE: T5F17.110 [At4g28660] |14149922..14151103 |SUPPORT | 2 exon(s) |PROD: photosystem II protein W - like |5"-UTR: 92 | -500: +1 | +1 ... CDS sta

Length of Query Sequence: 500 bp | Nucleotide Frequencies: A - 0.29 G - 0.18 T - 0.29 C - 0.23

............................................................

RE: 175. AC: RSP00175//OS: arabidopsis (Arabidopsis thaliana) /GENE: synthetic oligonucleotides/RE: ABRE /BF: ABFs

Found in 8 ( 80.00 %) SEQs (out of 10)

Motifs on "-" Strand: Mean Exp. Number 0.00325 Found 1

214 CACGTGGC 207 (Mism.= 0)

............................................................

RE: 204. AC: RSP00204//OS: arabidopsis (Arabidopsis thaliana) /GENE: AtEm6/RE: ABRE/6.2 /BF: ABI5

Found in 10 (100.00 %) SEQs (out of 10)

Motifs on "-" Strand: Mean Exp. Number 0.00488 Found 1

216 cACACGTGGC 207 (Mism.= 1)

............................................................

RE: 524. AC: RSP00524//OS: carrot (Daucus carota) /GENE: Dc3/RE: E4-core /BF: DPBF-1; DPBF-2;

Found in 8 ( 80.00 %) SEQs (out of 10)

Motifs on "-" Strand: Mean Exp. Number 0.02215 Found 1

215 ACACGTG 209 (Mism.= 0)

............................................................

RE: 723. AC: RSP00723//OS: tobacco (Nicotiana plumbaginifolia) /GENE: rbcS 8B/RE: G-box /BF: HY5

Found in 8 ( 80.00 %) SEQs (out of 10)

Motifs on "-" Strand: Mean Exp. Number 0.00325 Found 1

214 CACGTGGC 207 (Mism.= 0)

............................................................

RE: 1041. AC: RSP01034//OS: arabidopsis (Arabidopsis thaliana) /GENE: RD29B/RE: ABRE 1/2 /BF: ABI3; ABI5; AREB1

Found in 8 ( 80.00 %) SEQs (out of 10)

Motifs on "-" Strand: Mean Exp. Number 0.01788 Found 1

213 ACGTGGC 207 (Mism.= 0)

............................................................

RE: 1602. AC: RSP01570//OS: rice (Oryza sativa, indica) /GENE: Synthetic oligonucleotides/RE: Em1a /BF: OSBZ

Found in 8 ( 80.00 %) SEQs (out of 10)

Motifs on "-" Strand: Mean Exp. Number 0.00325 Found 1

214 CACGTGGC 207 (Mism.= 0)

............................................................

RE: 1850. AC: RSP01816//OS: tobacco (Nicotiana tabacum) /GENE: PNZIP/RE: G-box /BF: NtbZIP

Found in 10 (100.00 %) SEQs (out of 10)

Motifs on "+" Strand: Mean Exp. Number 0.00318 Found 1

207 GCCACGTGTg 216 (Mism.= 1)

............................................................

RE: 2866. AC: RSP02832//OS: foxtail millet (Setaria italica) /GENE: SiARDP/RE: ABRE2 /BF: SiAREB1 ; SiAREB2

Found in 8 ( 80.00 %) SEQs (out of 10)

Motifs on "-" Strand: Mean Exp. Number 0.01788 Found 1

213 ACGTGGC 207 (Mism.= 0)

............................................................

In total 8 motifs from 8 different REs have been found.

------------------------------------------------------------

9. QUERY: > EXAMPLE Group (10 genes) | Arabidopsis, Chr 4: GENE: F16A16.140 [At4g28750] |complement(14202779..14203961) |SUPPORT | 3 exon(s) |PROD: photosystem I subunit PSI-E - like protein |5"-UTR: 67 | -5

Length of Query Sequence: 500 bp | Nucleotide Frequencies: A - 0.42 G - 0.12 T - 0.30 C - 0.16

............................................................

RE: 175. AC: RSP00175//OS: arabidopsis (Arabidopsis thaliana) /GENE: synthetic oligonucleotides/RE: ABRE /BF: ABFs

Found in 8 ( 80.00 %) SEQs (out of 10)

Motifs on "+" Strand: Mean Exp. Number 0.00046 Found 1

335 CACGTGGC 342 (Mism.= 0)

............................................................

RE: 204. AC: RSP00204//OS: arabidopsis (Arabidopsis thaliana) /GENE: AtEm6/RE: ABRE/6.2 /BF: ABI5

Found in 10 (100.00 %) SEQs (out of 10)

Motifs on "+" Strand: Mean Exp. Number 0.00081 Found 1

333 tACACGTGGC 342 (Mism.= 1)

............................................................

RE: 524. AC: RSP00524//OS: carrot (Daucus carota) /GENE: Dc3/RE: E4-core /BF: DPBF-1; DPBF-2;

Found in 8 ( 80.00 %) SEQs (out of 10)

Motifs on "+" Strand: Mean Exp. Number 0.00989 Found 1

334 ACACGTG 340 (Mism.= 0)

............................................................

RE: 723. AC: RSP00723//OS: tobacco (Nicotiana plumbaginifolia) /GENE: rbcS 8B/RE: G-box /BF: HY5

Found in 8 ( 80.00 %) SEQs (out of 10)

Motifs on "+" Strand: Mean Exp. Number 0.00046 Found 1

335 CACGTGGC 342 (Mism.= 0)

............................................................

RE: 1041. AC: RSP01034//OS: arabidopsis (Arabidopsis thaliana) /GENE: RD29B/RE: ABRE 1/2 /BF: ABI3; ABI5; AREB1

Found in 8 ( 80.00 %) SEQs (out of 10)

Motifs on "+" Strand: Mean Exp. Number 0.00287 Found 1

336 ACGTGGC 342 (Mism.= 0)

............................................................

RE: 1602. AC: RSP01570//OS: rice (Oryza sativa, indica) /GENE: Synthetic oligonucleotides/RE: Em1a /BF: OSBZ

Found in 8 ( 80.00 %) SEQs (out of 10)

Motifs on "+" Strand: Mean Exp. Number 0.00046 Found 1

335 CACGTGGC 342 (Mism.= 0)

............................................................

RE: 1850. AC: RSP01816//OS: tobacco (Nicotiana tabacum) /GENE: PNZIP/RE: G-box /BF: NtbZIP

Found in 10 (100.00 %) SEQs (out of 10)

Motifs on "-" Strand: Mean Exp. Number 0.00052 Found 1

342 GCCACGTGTa 333 (Mism.= 1)

............................................................

RE: 2866. AC: RSP02832//OS: foxtail millet (Setaria italica) /GENE: SiARDP/RE: ABRE2 /BF: SiAREB1 ; SiAREB2

Found in 8 ( 80.00 %) SEQs (out of 10)

Motifs on "+" Strand: Mean Exp. Number 0.00287 Found 1

336 ACGTGGC 342 (Mism.= 0)

............................................................

In total 8 motifs from 8 different REs have been found.

------------------------------------------------------------

10. QUERY: > EXAMPLE Group (10 genes) | Arabidopsis, Chr 5: GENE: K1F13.25 [At5g66570] |26585884..26587504 |SUPPORT | 3 exon(s) |PROD: photosystem II oxygen-evolving complex 33 (OEC33) |5"-UTR: 86 | -500: +1

Length of Query Sequence: 500 bp | Nucleotide Frequencies: A - 0.33 G - 0.16 T - 0.29 C - 0.22

............................................................

RE: 175. AC: RSP00175//OS: arabidopsis (Arabidopsis thaliana) /GENE: synthetic oligonucleotides/RE: ABRE /BF: ABFs

Found in 8 ( 80.00 %) SEQs (out of 10)

Motifs on "+" Strand: Mean Exp. Number 0.00210 Found 1

331 CACGTGGC 338 (Mism.= 0)

............................................................

RE: 204. AC: RSP00204//OS: arabidopsis (Arabidopsis thaliana) /GENE: AtEm6/RE: ABRE/6.2 /BF: ABI5

Found in 10 (100.00 %) SEQs (out of 10)

Motifs on "+" Strand: Mean Exp. Number 0.00286 Found 1

329 GACACGTGGC 338 (Mism.= 0)

............................................................

RE: 524. AC: RSP00524//OS: carrot (Daucus carota) /GENE: Dc3/RE: E4-core /BF: DPBF-1; DPBF-2;

Found in 8 ( 80.00 %) SEQs (out of 10)

Motifs on "+" Strand: Mean Exp. Number 0.01965 Found 1

330 ACACGTG 336 (Mism.= 0)

............................................................

RE: 723. AC: RSP00723//OS: tobacco (Nicotiana plumbaginifolia) /GENE: rbcS 8B/RE: G-box /BF: HY5

Found in 8 ( 80.00 %) SEQs (out of 10)

Motifs on "+" Strand: Mean Exp. Number 0.00210 Found 1

331 CACGTGGC 338 (Mism.= 0)

............................................................

RE: 1041. AC: RSP01034//OS: arabidopsis (Arabidopsis thaliana) /GENE: RD29B/RE: ABRE 1/2 /BF: ABI3; ABI5; AREB1

Found in 8 ( 80.00 %) SEQs (out of 10)

Motifs on "+" Strand: Mean Exp. Number 0.00947 Found 1

332 ACGTGGC 338 (Mism.= 0)

............................................................

RE: 1159. AC: RSP01151//OS: maize (Zea mays) /GENE: Em/RE: Em1a /BF: EmBP-1 (+VP1)

Found in 8 ( 80.00 %) SEQs (out of 10)

Motifs on "+" Strand: Mean Exp. Number 0.00228 Found 1

329 GACACGTGGC 338 (Mism.= 0)

............................................................

RE: 1602. AC: RSP01570//OS: rice (Oryza sativa, indica) /GENE: Synthetic oligonucleotides/RE: Em1a /BF: OSBZ

Found in 8 ( 80.00 %) SEQs (out of 10)

Motifs on "+" Strand: Mean Exp. Number 0.00210 Found 1

331 CACGTGGC 338 (Mism.= 0)

............................................................

RE: 1850. AC: RSP01816//OS: tobacco (Nicotiana tabacum) /GENE: PNZIP/RE: G-box /BF: NtbZIP

Found in 10 (100.00 %) SEQs (out of 10)

Motifs on "-" Strand: Mean Exp. Number 0.00189 Found 1

338 GCCACGTGTC 329 (Mism.= 0)

............................................................

RE: 2866. AC: RSP02832//OS: foxtail millet (Setaria italica) /GENE: SiARDP/RE: ABRE2 /BF: SiAREB1 ; SiAREB2

Found in 8 ( 80.00 %) SEQs (out of 10)

Motifs on "+" Strand: Mean Exp. Number 0.00947 Found 1

332 ACGTGGC 338 (Mism.= 0)

............................................................

In total 9 motifs from 9 different REs have been found.

------------------------------------------------------------
