# Supplementary material for: Nsite, NsiteH and NsiteM computer tools for studying transcription regulatory elements
Source: Bioinformatics. 2015 Jul 2;31(21):3544–5. doi: 10.1093/bioinformatics/btv404 (PMC4612222; doi:10.1093/bioinformatics/btv404)
Supplement: Supplementary Data [file supp_btv404_BNotes_NSITE_Sovovyev_Supplementary_3.docx]

**Statistical approach applied in NSITE programs**

**Rationale of the approach**

1. Regulatory element (RE) may consist of a single box (a continuous DNA segment) or two boxes, spaced by some DNA sequence, where only its length, but not the nucleotide content, of this spacer is important for functioning of such a composite site.
2. A real RE or its IUPAC consensus contains both variable positions (where the presence of a certain group of nucleotides is acceptable ), and strictly conserved positions (where a strong identity between real site/consensus and predicted motif is required). The nonequivalence of these positions should be taken into account, i.e., complete identity at conserved positions is required, and mismatches in the variable positions should be allowed.
3. The similarity between the RE and a motif in a query DNA sequence may occur by chance (especially for short motifs), therefore estimation of its statistical significance is of major importance.
4. When describing a consensus we do not use its nucleotide frequencies because of small sizes of regulatory motifs. Instead, we base our estimations on the number of nucleotides of various types in the consensus.
5. Although all available databases on REs usually present a fixed distance between two boxes of composite elements, some variability of the spacer length seems, to have place. Therefore, a search algorithm for composite REs should allow some limited flexibility in the spacer length relying on both the known experimental data and theoretical assumptions.

**Statistical estimation of putative RE motifs**

Let us assume that we are searching for a site in a sequence of length *N* with random arrangement of nucleotides A, T(U), G and C, where the frequencies of these nucleotides are *P_A_, P_T(U)_, P_G_* and *P_C_*, respectively. If we accept that *P_1_=P_A,_, P_2_=P_G_, P_3_=P_T_, P_4_=P_C_*, then the frequencies of the nucleotides of the other classes *P_j_ (j=5,…, 15)* are determined as sums of frequencies of nucleotides of all the types in the *j*-th class.

***Simple (one - block) site.*** Let us consider the site of length *L* characterized by the values *N_l_ (l=1,…15)*, where *N_l_* is the number of nucleotides of the *l*-th class belonging to the site and *N_1_ + N_2_ + …+ N_15_ = L*.

Let the site has *M* conserved positions, where *M_l_ (l=1,…,14)* is the number of conserved nucleotides of *l*-th class (*M_1_ + M_2_ + …+ M_14_ = M*). Then *k (k=0,1,…)* mismatches between the site and the segment of length *L* belonging to the sequence under consideration are allowed only at *L-M* variable positions. *R_l_* *(l=1,…,14)* mismatches between the consensus and the DNA segment for the *l*-th class of nucleotides meet the following conditions: 0≤*R_1_*≤min *(k, N_1_-M_1_),* 0≤*R_2_<*min *(k-R_1_, N_15_­-M_15_),…,* 0≤*R_15_<*min *(k-R_1_-R­_2_-…-R_13_, N_14_­-M_14_)*.

Under assumption of the binomial distribution of matches and mismatches, the probability *P(L,k)* of detecting the motif *(L,k)* of length *L* different in *k* variable positions from the consensus is:

(1)

In this case the expected number of such motifs *(L, k)* in a random sequence of length *N* is

where *FL = N-L+1*. The probability of having precisely T motifs *(L, k)* in the sequence may be estimated using binomial distributions:

(2)

The probability of detecting T motifs having not more than *k* differences from the consensus is:

(3)

Now we can derive the upper boundary of the confidence interval *T_0_* (with the significance level *q*) for the expected number of motifs in the random sequence:

and (4)

If the number of motifs *(L, k)* detected in the real sequence meets the condition *T≥T_0_*, we consider one or a group of motifs as non-random, with significance level *q*.

***Composite (two - blocks) site.*** Let us consider a composite site containing two blocks of lengths *L_1_* and *L_2_* at a distance *D* apart *(D1­<D<D_2_,* i.e., *D_1_* and *D_2_* are, respectively, the minimum and maximum allowed distances between the blocks). Let *N_1l_* and *N_2l_* be the number of nucleotides of the *l*-th class in the first and second blocks, respectively (*l=*1,…,15*)*. It is clear that *N_j1_ + N_j2_ + …+ N_j15_ = L_j_ (j=1,2).*

Let the first and second blocks have *M_1l­_* and *M_2l_* conserved positions of the nucleotides of *l*-th class. Then the probability *P(L_j_, k_j_)* of finding the motif *(L_j_,k_j_)* of size *L_j_* with *k_j_* mismatches from the *j*-th block of the site is calculated using Eq. (2) with the substitutions *L, k, N_l_* and *M_l_* by *L_j_, k_j_, N_jl_* and *M_jl_ (l=*1,…,14; *j=*1,2), respectively. The probability of occurrence of both motifs *(L_1_, k_1_)* and *(L_2_, k_2_)* in the random sequence is:

*P(L_1_, k_1_,; L_2_, k_2_)=P(L_1_, k_1_)*×*P(L_2_, k_2_)*. (5)

The number of possible ways of arranging the segments *(L_1_, k_1_)* and *(L_2_, k_2_)* in the random sequence of length *N* (under the condition that *D_1_<D<D_2_)* is:

(6)

Thus, the expected number of composite motifs *(L_1_, k_1_,; L_2_, k_2_, D_1_, D_2_)* is:

*(L_1_,k_1_,L_2_,k_2_,D_1_,D_2_) = F(L_1_,L_2_,D_1_,D_2_)*×*P(L_1_, k_1_,; L_2_, k_2_)* (7*)*

The probability *P(T)* of detecting *T* composite motifs *(L_1_, k_1_,; L_2_, k_2_, D_1_, D_2_)* in the random sequence is determined using Eqs. (2) and (3) with *F* and *P(L,k)* substituted by *F(L_1_,L_2_,D_1_,D_2_)* and *P(L_1_, k_1_,; L_2_, k_2_*) given in Eqs. (5) and (6). At last, using the obtained values *P(T),* the upper boundary of the confidence interval *T_0_*  is determined from conditions (4). We assume occurrence of nonrandom similarity between the composite consensus and the DNA sequence segments, if *T≥T_0_* .
