# Supplementary material for: Nsite, NsiteH and NsiteM computer tools for studying transcription regulatory elements
Source: Bioinformatics. 2015 Jul 2;31(21):3544–5. doi: 10.1093/bioinformatics/btv404 (PMC4612222; doi:10.1093/bioinformatics/btv404)
Supplement: Supplementary Data [file supp_btv404_BNotes_NSITE_Sovovyev_Supplementary_4.docx]

**Search algorithms implemented in Nsite, NsiteH and NsiteM programs:**

**

**

**Fig. S6.** The flow-charts of algorithms of search for statistically Nonrandom Motifs (NRMs) of known regulatory elements (REs) implemented in Nsite, Nsite_h and Nsite_M programs..

**Nsite.** The input to the program may consist of a single or multiple query sequences in FASTA format. Search for NRMs includes the following steps.

1. For every RE of length L, listed in the data file of REs (option “-d:’), compute maximum allowed number of mismatches (*k*):

*k = L*(100 – H),*

where *H* is a given homology level between the RE and its motif; 0 ≤ H ≤ 100.

1. For every known RE compute the expected number *T_e_(l,k)* of such motifs *(L,k)* in a random sequence of the same length and nucleotide composition as the query sequence and the upper boundary of the confidence interval *T_0_* with a given significance level *q* (see: Supplementary File 3).
2. For every known RE, search for motifs with mismatches ≤ k.
3. If the number (T) of the motifs for some RE found in the real sequence meets the conditions *T≤T_e_(l,k)* and *T≥T_0_*, we consider these motifs as NRMs of the RE with significance level *q*.
4. Output NRMs (see: Fig. S2, Supplementary File 1.
5. If Input file contains multiple query sequences, then go to analyze the next query.

**NsiteH. The** input to the program consists of 2 orthologous or homologous query sequences in separate FASTA format files. Search for NRMs includes the following steps.

1. For every RE of length L, listed in the data file of REs (option “-d:’), compute maximum allowed number of mismatches (k):

*k = L*(100 – H),*

where *H* is a given homology level between the RE and its motif; 0 ≤ H ≤ 100.

1. Separately, for every query sequence and for every known RE, compute the expected number *T_e_(l,k)* of such motifs *(L,k)* in a random sequence of the same length and nucleotide composition as the query sequence and the upper boundary of the confidence interval *T_0_* with a given significance level *q* (as in case of **Nsite**).
2. Align query sequences by SCAN2 program (<http://softberry.com/scan.html>); example of the alignment results is presented in Fig. S7.
3. In both query sequences, for every known RE, search for motifs with mismatches ≤ k.


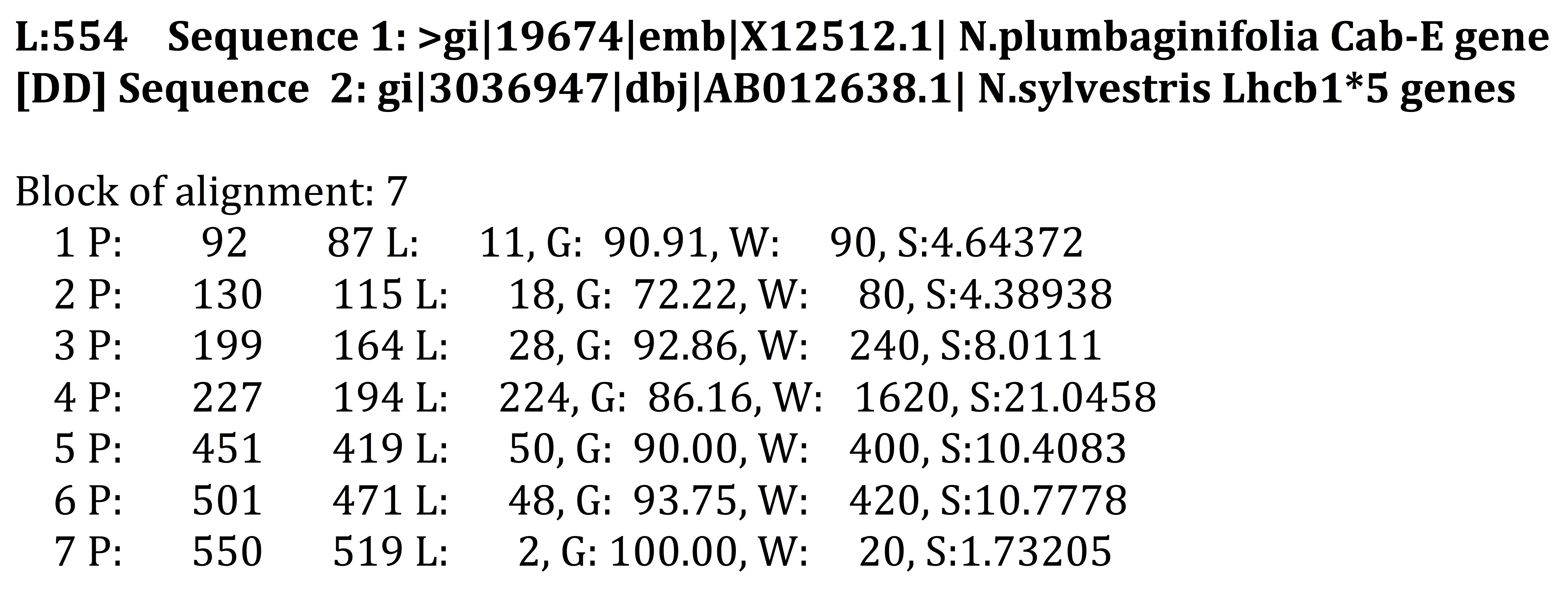


**Fig. S7**. Output results of SCAN2 program (<http://softberry.com/scan.html>) for alignment of 2 query sequences. The lines from “1 P:” to “7 P:” start positions if similarity region in Query 1 and Query2 sequences (e.g. 92 and 87, respectively), length of similarity region (e.g. 11) and level of similarity of these regions (e.g. 90,91%).

1. For every query, if the number (T) of the motifs for some RE found in the real sequence meets the conditions *T≤T_e_(l,k)* and *T≥T_0_*, we consider these motifs as NRMs of the corresponding RE with significance level *q*.
2. For both query sequences, using the SCAN2 alignment data, detect NRMs with conservation level equal to or higher a given homology level (option: “-s:”), referred as conservative NRMs.
3. Output conservative NRMs (see: Fig. S3, Supplementary File 1).

**NsiteM. The** input to the program consists of two ore more query sequences in a single FASTA format file. Search for NRMs includes the following steps.

1. For every RE of length L, listed in the data file of REs (option “-d:’), compute maximum allowed number of mismatches (k):

*k = L*(100 – H),*

where *H* is a given homology level between the RE and its motif; 0 ≤ H ≤ 100.

1. Separately, for every query sequence and for every known RE, compute the expected number *T_e_(l,k)* of such motifs *(L,k)* in a random sequence of the same length and nucleotide composition as the query sequence and the upper boundary of the confidence interval *T_0_* with a given significance level *q* (as in case of **Nsite**).
2. In each query sequences, for every known RE, search for motifs with mismatches ≤ k_0_.
3. For each query, if the number (T) of the motifs for some RE found in the real sequence meets the conditions *T≤T_e_(l,k)* and *T≥T_0_*, we consider these motifs as NRMs of the RE with significance level *q*.
4. If NRMs of the same RE found in a given portion (option: “-t:”) or more query sequences, these NRMs are selected and presented in the output of the program.
5. Selected NRM outputted as a graph, as well as a list of motifs with their detailed characteristics (see: Fig. S4, Supplementary File 1).
